# Gene expression profiling of *Spodoptera frugiperda *hemocytes and fat body using cDNA microarray reveals polydnavirus-associated variations in lepidopteran host genes transcript levels

**DOI:** 10.1186/1471-2164-7-160

**Published:** 2006-06-21

**Authors:** M Barat-Houari, F Hilliou, F-X Jousset, L Sofer, E Deleury, J Rocher, M Ravallec, L Galibert, P Delobel, R Feyereisen, P Fournier, A-N Volkoff

**Affiliations:** 1 UMR 1231 Biologie Intégrative et Virologie des Insectes. INRA – Université de Montpellier II. Place Eugène Bataillon, Case Courrier 101, 34 095 Montpellier Cedex, France; 2 UMR 1112 R.O.S.E. INRA – Université de Nice-Sophia Antipolis, Laboratoire de Génomique Fonctionnelle des Insectes, 400 route des Chappes, BP 167, 06 903 Sophia Antipolis Cedex, France; 3 INRA U.M.R. Sciences pour l'Oenologie, Equipe Microbiologie – Bât 28, 2, place Viala, 34 060 Montpellier Cedex 01, France

## Abstract

**Background:**

Genomic approaches provide unique opportunities to study interactions of insects with their pathogens. We developed a cDNA microarray to analyze the gene transcription profile of the lepidopteran pest *Spodoptera frugiperda *in response to injection of the polydnavirus HdIV associated with the ichneumonid wasp *Hyposoter didymator*. Polydnaviruses are associated with parasitic ichneumonoid wasps and are required for their development within the lepidopteran host, in which they act as potent immunosuppressive pathogens. In this study, we analyzed transcriptional variations in the two main effectors of the insect immune response, the hemocytes and the fat body, after injection of filter-purified HdIV.

**Results:**

Results show that 24 hours post-injection, about 4% of the 1750 arrayed host genes display changes in their transcript levels with a large proportion (76%) showing a decrease. As a comparison, in *S. frugiperda *fat body, after injection of the pathogenic JcDNV densovirus, 8 genes display significant changes in their transcript level. They differ from the 7 affected by HdIV and, as opposed to HdIV injection, are all up-regulated. Interestingly, several of the genes that are modulated by HdIV injection have been shown to be involved in lepidopteran innate immunity. Levels of transcripts related to calreticulin, prophenoloxidase-activating enzyme, immulectin-2 and a novel lepidopteran scavenger receptor are decreased in hemocytes of HdIV-injected caterpillars. This was confirmed by quantitative RT-PCR analysis but not observed after injection of heat-inactivated HdIV. Conversely, an increased level of transcripts was found for a galactose-binding lectin and, surprisingly, for the prophenoloxidase subunits. The results obtained suggest that HdIV injection affects transcript levels of genes encoding different components of the host immune response (non-self recognition, humoral and cellular responses).

**Conclusion:**

This analysis of the host-polydnavirus interactions by a microarray approach indicates that the presence of HdIV induces, directly or indirectly, variations in transcript levels of specific host genes, changes that could be responsible in part for the alterations observed in the parasitized host physiology. Development of such global approaches will allow a better understanding of the strategies employed by parasites to manipulate their host physiology, and will permit the identification of potential targets of the immunosuppressive polydnaviruses.

## Background

**Figure 1:**
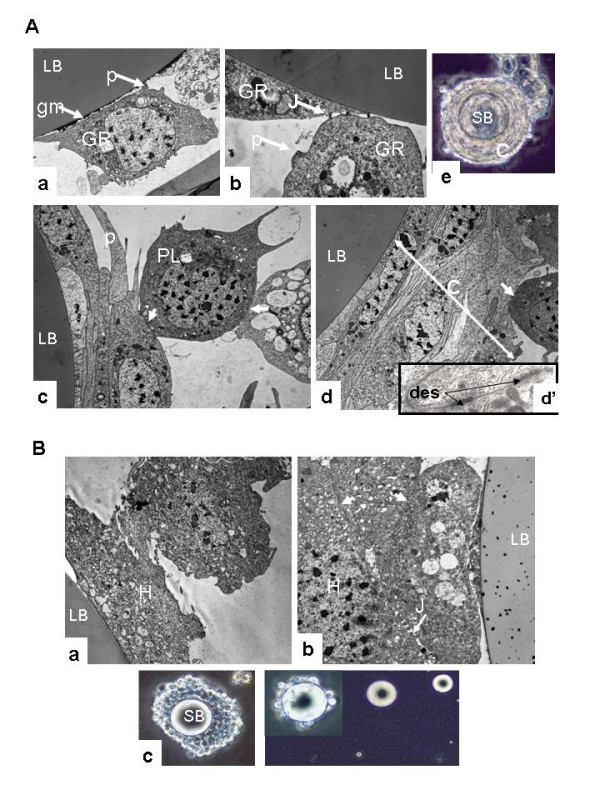
Encapsulation of beads in *Spodoptera frugiperda *last instar larvae. **A. **Time course of encapsulation, by transmission electronic and photonic observations. **a) **Bead 30 minutes post-injection (p.i.). Beads are covered by an uncharacterized granular material (as described in *Galleria mellonella *by Schmit and Ratcliffe [115]). **b) **Bead 1 hour p.i. **c) **Bead 2 hours p.i. **d) **Bead 4 hours p.i. **d') **Detail showing desmosomes between two cells involved in the capsule. **e) **Bead 24 hours p.i. (phase contrast observation). **B. **Effect of injection of HdIV on cellular response. In a) and b) HdIV have been injected 5 hours prior to the beads, in c) HdIV and beads were injected simultaneously. **a) **Bead 15 hours p.i. **b) **Bead 6 hours p.i. **c) **Beads 24 hours p.i. (phase contrast observation). LB: latex bead; C: capsule; gm: granular material; des: desmosome-like junction; H: hemocyte; J: cellular junction; p: pseudopode-like extension; SB: sephadex bead; arrowheads indicate cells already partially attached to each other. The scale bar corresponds to 1 μm in all views, with exception of Figure 1d'), where the scale bar corresponds to 0.5 μm.

Unlike mammals, the defense against microorganisms and foreign organisms in insects relies exclusively on the innate immune response composed of complex and interconnected humoral and cellular mechanisms [1,2]. The humoral response consists of the synthesis of a large variety of antimicrobial peptides (AMPs) mainly by the fat body cells (the equivalent of the liver in mammals) and proteolytic cascades which, upon activation, lead to blood coagulation or melanization [3-6]. Cellular responses include phagocytosis of invading bacteria, apoptotic bodies or small abiotic targets, and the formation of capsules around larger invading intruders such as parasitic wasps' eggs [7]. Lastly, insect antiviral response is still poorly understood but recent studies demonstrate the increasing interest raised by this response [8-10]. The immune response is well known for dipteran insects such as flies and mosquitoes and a large amount of data is also available for lepidopteran insects. In the latter, several AMPs have been characterized. Signal transduction pathways leading to their transcription are probably similar to those of *Drosophila*, since regulatory motifs such as the kappaB-like and GATA sequences have been identified [11-13] and transcriptional induction by immune challenge has been reported [14]. Pattern recognition proteins such as hemolin, peptidoglycan recognition protein, beta-1,3-glucan recognition proteins and immulectins have also been described in lepidopteran insects [15]. Regarding antiviral response within the insect hemocoel in lepidopteran insects, recent studies suggest involvement of humoral effectors such as prophenoloxydase [16] or hemolin [17] and of a cell-mediated response [18]. An increasing number of studies focus on the lepidopteran cellular response and several effectors, including a cytokine-like, receptors or cellular adhesion molecules, have been identified [2,5,19-22]. Encapsulation is a rapid event that results from the activity of hemocytes capable of adhering to invading foreign organisms, the granulocytes and plasmatocytes [2,22,23]. In *S. frugiperda*, half an hour after their injection into last instar larvae, hemocytes are already binding to the latex beads (Figure 1A, a). The subsequently recruited hemocytes adhere to the one already spread on the bead in successive layers (Figure 1A, b and 1c) and the capsule, with abundant desmosome-like structures, is complete around most of the beads 8 hours after injection (Figure 1A, d and 1d').

In parasitic wasps, various mechanisms have evolved to circumvent the host immune response and allow successful pre-imaginal development [24-28]. Some species, such as the endoparasitoid wasp *Hyposoter didymator *from the *Ichneumonidae *family, utilize endosymbiotic viruses of the Polydnavirus family, characterized by circular segmented DNA genomes, which protect the developing wasp against host defenses. The *Hyposoter didymator *ichnovirus (HdIV) particles are injected along with the egg into the hemolymph of the lepidopteran host *Spodoptera frugiperda *larvae. Viral replication occurs exclusively in the calyx of the wasp ovary and high concentrations of viral particles are produced [29]. When introduced into the lepidopteran host hemocoel, HdIV infects several host tissues and the HdIV genes that have been studied to date are rapidly and consistently transcribed [30-33]. Expression of viral genes is associated with phenotypic variations resulting from physiological and developmental alterations in the parasitized larvae, which are required for successful parasitoid development. Among these physiological alterations, HdIV inhibits host humoral and cellular immunity. Indeed, when HdIV is injected 5 hours before or simultaneously with latex beads, the latter are no longer encapsulated (Figure 1B). However, *S. frugiperda *hemocytes conserve the ability to adhere to the bead and to each other (Figure 1B). HdIV-induced impaired encapsulation is due in part to disruption of the actin cytoskeleton (Volkoff, pers. data), as observed in several other systems [34,35]. Another effect of parasitism by polydnavirus-associated wasps is a reduction of prophenoloxidase (proPO) activity and of plasma melanization [36-41]. PO plays a role in cuticle sclerotization, wound healing, and sequestering/killing of invading pathogens (reviewed in [42]). The question as to why it is advantageous for the parasitic wasp or the virus to inhibit proPO activation remains unresolved.

Thus, polydnaviruses are responsible for the disruption of various insect immune components (reviewed in [24]). However, little is known about the precise molecular mechanisms involved. Recent polydnavirus sequencing projects allowed the discovery of a large panel of candidates as virulence or immune suppressive factors, which are encoded by the segmented DNA genomes [43-45]. These polydnavirus genomes, including that of HdIV, encode a family of genes with homology with the *Drosophila *ankyrin-repeat Cactus protein, the inhibitor of NF-κB in the Toll pathway [43,46]. Some of these viral inhibitor kappaB-like proteins have been recently shown to be potent inhibitors of the insect immune system by targeting the NF-κB pathway [47]. With the exception of the viral ankyrins and the viral innexins [48], none of the genes identified until now in the HdIV genome have significant similarities with known genes. Therefore, despite increasing available information on polydnavirus gene sequences, little is known to date about the host cellular targets of the viral gene products. Parasitism often dramatically affects levels of host hemolymph proteins or enzymes (growth-associated proteins such as arylphorin, riboflavin binding hexamer or juvenile hormone esterase, or antibacterial molecules such as lysozyme and cecropin) [49-55]. These changes can be explained by consumption of hemolymph by the parasitoid larva or can arise from alterations in levels of gene transcription [51], protein synthesis or other post-transcriptional modifications [49,52-55]. However, reports indicate that gene transcript levels are not changed by polydnavirus infection [53-55] or by calyx fluid injection [51]. Thus, there is no data today that demonstrate regulation of host transcripts by polydnaviruses or calyx fluid.

In insects, microarray approaches have previously been used for analysis of host-pathogen interactions (for example, for *Drosophila*: [8,56,57]; for *Anopheles*: [58]) or host-parasite interactions (for *Drosophila*: [9,59]; for *Anopheles*: [60]). These studies generally pertain to model insects for which genomic tools are available. However, none of these well-known models are hosts for parasitic wasps associated with polydnaviruses, since this family of viruses is reported only from larval endoparasitoids that develop on lepidopteran hosts, and to our knowledge no such parasitoid attacks the silkworm *Bombyx mori*, a model lepidopteran with a fully sequenced genome. Thus, because of lack of available tools, such global analyses have not yet been undertaken with lepidopteran species hosting polydnavirus-associated parasitic wasps.

The present study was aimed at investigating by a microarray approach whether HdIV injection induces modifications in the levels of *S. frugiperda *gene transcript levels, resulting either directly from viral gene products or from upstream regulations or feed-back mechanisms. Analysis of the nature of the host genes displaying modified transcript levels, if any, may thus provide clues on the pathways targeted by polydnaviruses and associated factors during parasitism. Moreover, since we are dealing with an immunosuppressive agent, some of those genes may represent new candidate molecules involved in the lepidopteran immune response.

In this study we aimed to establish the gene expression profile of the two main effectors of the *S. frugiperda *immune response, the fat body and the hemocytes, 24 hours after injection of the polydnavirus HdIV. We report in this paper that transcript levels for several host genes are indeed modified in the two immune tissues following HdIV injection. As discussed below, a number of these genes correspond to genes previously shown to be involved in lepidopteran innate immunity.

## Results and discussion

A preliminary construction of four EST libraries for the noctuid pest *S. frugiperda *(EST sequences deposited in the database Spodobase: [61]) allowed us to design a microarray comprising triplicates of 1750 cDNA PCR products printed on glass slides. This cDNA microarray was used to analyze the transcriptional changes in *S. frugiperda *tissues 24 hours after injection of filter-purified HdIV. At this chosen time point, the HdIV genes studied so far are all transcribed in the lepidopteran host [30-33]. Ten viral cDNAs were spotted on the microarray. Among these genes, four (D8, K29, M24 and P30) displayed significant increases in transcript levels in *S. frugiperda *hemocytes and two (M24 and P30) in the fat body (Additional file 1A). These results were consistent with previous Northern-blot analyses showing high levels of transcripts for these viral genes 24 hours post injection (pi) [30,32].

**Figure 2:**
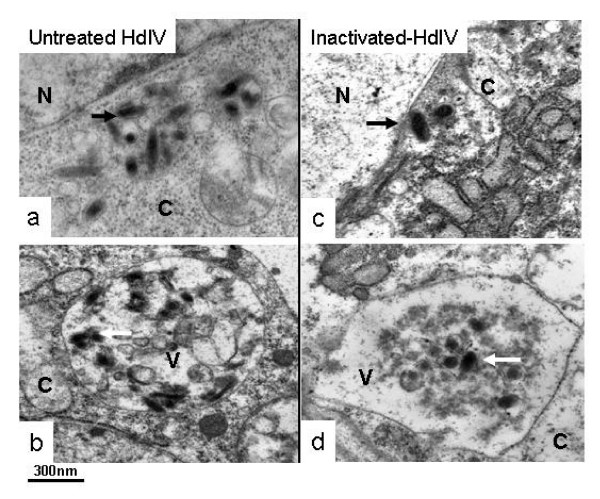
Infection of *Spodoptera frugiperda *hemocytes after infection of untreated HdIV (left panels, a and b) and heat-inactivated HdIV (right panels, c and d) by transmission electronic observations. For both control and heat treatment, HdIV particles can be observed within the cytoplasm of hemocytes (black arrows, a and c) and in vacuoles (white arrows, b and d). C: cytoplasm; N: nucleus; V: vacuole.

After microarray analyses, a subset of responsive *S. frugiperda *genes was selected and their transcript levels were analyzed by quantitative RT-PCR 24 hours after injection of heat-inactivated HdIV. Heat inactivation resulted in the absence of viral gene transcription, which was verified by RT-PCR for the viral innexin-1 gene, but did not impair entrance of heat-treated viral particles in host hemocytes (HdIV particles were found in the cytoplasm as well as in vacuoles, similarly to non-heated HdIV; Figure 2). We expected this assay to provide clues whether the observed variations in gene transcript levels were related to HdIV gene transcription or were only the result of the presence of virus particles (recognition).

Moreover, to ascertain that microarray results were relevant to HdIV, and did not merely reflect a global response of the lepidopteran host to the presence of an invader of small size such as a virus, we also analyzed the transcriptional profile of *S. frugiperda *fat body 24 hours after injection of the *Junonia coenia *densovirus (JcDNV). JcDNV is a highly pathogenic non-enveloped virus for *S. frugiperda *larvae that presents a wide range of tissue tropism, including hemocytes and fat body, the latter being the main target organ [62].

### General overview of transcriptional profiles in *S. frugiperda *after injection of HdIV

As shown in Table 1, 18 genes are significantly up-regulated and 54 genes are significantly down-regulated in *S. frugiperda *hemocytes, 24 hours after injection of filter-purified HdIV. This represents 1.4 % and 3.7 % of the arrayed cDNAs, respectively (*q-value *= 0.0056; they correspond to a total of 24 and 65 spots, respectively; see Additional file 1B). The *S. frugiperda *transcripts increase up to 5.9-fold for the up-regulated ones (56% range from 1.6 to 2.7 and 44% from 3.0 to 5.9), and decrease up to 3.8-fold for the down-regulated ones (61% from -1.5 to -1.9 and 39% from -2.0 to -3.8). In the HdIV-infected fat body, using both SAM and GeneAnova analyses, we detected only 7 transcripts (0.40 % from the overall arrayed cDNAs) that were significantly modulated, all negatively (*q-value *= 0.077). Five genes were down-regulated in both hemocytes and fat body (alpha and beta tubulins, enolase, deoxyribose-phosphate aldolase-like and calreticulin; Table 1). Fold changes in fat body are similar to those observed in the hemocytes (up to -2.6). A quantitative RT-PCR analysis corroborated the microarray results in HdIV-infected hemocytes and fat body for a subset of 8 genes putatively implicated in insect immunity (Table 2). After injection of heat-inactivated HdIV, 5 out of the 8 analyzed genes did not display any significant changes in their transcript level and 2 varied in the opposite direction when compared to the results of injection of non-inactivated HdIV (galectin and lysozyme; Table 2). These results suggest that for these genes, the variations in transcript level probably resulted from the transcription of HdIV genes.

The transcript levels of 8 genes (0.51 % of the arrayed cDNAs) displayed a significant increase (up to 5.8-fold) in *S. frugiperda *fat body 24 hours after injection of the densovirus JcDNV. Genes responsive after JcDNV injection differed from those modified after HdIV injection (Table 1). As opposed to HdIV injection, no gene was significantly down-regulated. This result strongly suggests that the transcriptional variations observed in HdIV-injected samples are associated with HdIV rather than merely reflect a response of the lepidopteran host to injection of small foreign invaders such as viruses. However, when the transcript levels of 8 selected genes were analyzed in the hemocytes, we found 2 genes, galectin and immulectin-2 that were similarly increased and decreased, respectively, by HdIV and JcDNV injection (Table 2). Injection of heat-inactivated HdIV led to a decrease of *galectin *transcript level and did not affect significantly *immulectin *(Table 2). This suggests that variations do not reflect *S. frugiperda *response to injection of viral particles but rather that these two genes are targeted by both HdIV and JcDNV.

Thus, both microarray and quantitative RT-PCR results indicate that HdIV injection affects *S. frugiperda *gene transcript levels. Whether these effects are direct or not remains to be further investigated. The expression of the gene encoding the storage protein arylphorin was not affected in our experiments, in agreement with previous reports [49,54]. Considering the hemocyte and fat body tissues, 4% of the arrayed genes displayed modified transcript levels following HdIV injection, the majority (76%) of them being down-regulated (Table 1). Whereas classical immune challenges cause the majority of the genes to be induced or up-regulated, our finding that genes are essentially down-regulated by HdIV injection was expected, since HdIV represses immune response and development.

### Several of the *S. frugiperda *down-regulated genes 24 hours after injection of HdIV encode proteins related to the immune response

In the hemocytes, 24 genes (44% of the down-regulated genes) have either no significant similarity with sequences deposited in databases or similarity with hypothetical proteins of unknown function (classes EII and EIII, as defined in Additional file 2). Based on the hypothesis that host targets for polydnaviruses and associated factors may represent key molecules in lepidopteran physiology, these novel molecules should be further investigated. Of particular interest are those that are prevalent in either one of the hemocyte or fat body libraries. For example two cDNAs that displayed the highest fold changes belong to large clusters in the hemocyte library (Sf1H00035-3-1 and Sf1H02709-3-1, with 19 and 38 clones, respectively, decreased 3.2 and 2.2 fold, respectively).

Among the 56 genes down-regulated in *S. frugiperda *hemocytes and fat body after HdIV injection, 32 have similarity with known proteins. At least 37% (12 genes belonging to classes AI, AII, AV, AVI and AIX; Table 1) have presumed ubiquitous functions in cell metabolism, but most of the others are potentially involved in different steps of insect immune responses, as discussed below.

#### Genes encoding proteins previously described as involved in the insect humoral response

##### Antimicrobial molecules

Two potential antimicrobial molecules, the cobatoxin-like (decrease 1.98-fold) and a c-type lysozyme (decrease 2.2-fold) have lower transcript levels in hemocytes 24 hours after injection of HdIV compared to injection of saline buffer. Conversely, lysozyme mRNA levels are higher 24 hours after injection of heat-inactivated HdIV, as measured by quantitative RT-PCR (Table 2), with higher fold changes in the fat body (+7.3) than in the hemocytes (+1.7). JcDNV injection also results in increased transcript levels in the fat body (+2.1 for cobatoxin and +1.7 for lysozyme). Cobatoxin is a cysteine-rich peptide that may be involved in antimicrobial defense [12] whereas lysozyme is a widely distributed ubiquitous enzyme involved in self-defense from bacterial infection [63]. In *Drosophila *adults, lysozyme genes are up-regulated 24 hrs after protozoan invasion and down-regulated after fungal invasion [9], but transcript levels remain unchanged after microbial infection [56], or infection with a picorna-like virus [9]. In lepidopteran insects, lysozyme genes are induced in both eggs and pre-imaginal instars in response to bacterial injection [14,37,64,65]. In *S. frugiperda*, our results indicate that the c-lysozyme mRNA is more abundant in fat body in response to inactivated-HdIV or to JcDNV compared to control. This suggests that transcript levels of this gene increased in response to viral presence. In host-parasitoid models involving polydnaviruses, the activity of host lysozyme is reported to be inhibited by parasitism [37]. In *Heliothis virescens *parasitized with *Campoletis sonorensis*, the observed reduced plasma lysozyme activity involves inhibition at a post-transcriptional step [53]. Our study is the first report of a transcriptional regulation of this antimicrobial gene due to polydnavirus presence and expression. Down-regulation could reflect an active way of protecting both parasitoid wasp and virus from host defense.

##### The pro-PO cascade

Our results indicate that HdIV down-regulates the proPO-activation system by directly interfering with transcript levels of genes encoding proteins involved in the enzymatic cascade. Indeed, in this study, we found that a *S. frugiperda *sequence (named *Sf_PPAE*), similar to the *Manduca sexta *proPO-activating enzyme PAP-1 gene [66], is specifically down-regulated in the hemocytes 24 hours after injection of HdIV particles (-2.2 in microarrays and -2.1 in quantitative RT-PCR). Similarly to the proPO-activating enzymes (PPAE), Sf_PPAE contains a carboxyl-terminal proteinase domain typical of the chymotrypsin family and has an amino-terminal regulatory "clip" domain (reviewed in [67]). Insect PO is synthesized as an active zymogen (proPO), and is activated by proteolytic cleavage, mediated by a proteinase cascade plus additional factors, including immulectins. The PPAEs are the terminal serine proteinases that carry out the proteolysis of the proPO precursor. In *M. sexta*, transcription of the proPO-activating enzyme PAP-1 gene is up-regulated in hemocytes and fat body in response to a bacterial challenge and down-regulated by treatment with 20-hydroxyecdysone, suggesting that this gene is under the dual control of immune and hormonal signals [68]. Our results show that HdIV infection down-regulates the transcript levels of the gene encoding this proteinase. Conversely, injection of heat-inactivated HdIV or of JcDNV did not affect transcript levels of *Sf_PPAE*. Down regulation of the *Sf_PPAE*, as well as of the immulectin gene (see below), should thus reduce the efficiency of pro-PO proteolysis, which could be linked to the inhibition of hemolymph melanization observed in HdIV-infected *S. frugiperda *caterpillars.

#### Genes encoding proteins putatively involved in the insect cellular immune response and/or non-self recognition

##### Immulectin

Immulectins are C-type lectins specific to Lepidoptera with a unique structure consisting of tandem carbohydrate recognition domains (CRDs). They function as pathogen recognition receptors in the innate immune system by activating proPO in hemolymph [69], and by participating in hemocyte nodule formation [70] and encapsulation [71,72]. HdIV infection down-regulates (-1.9 in microarrays and -4.5 in quantitative RT-PCR) a *S. frugiperda *gene with high similarity with the *M. sexta *immulectin-2 (AAF91316; Blast E value = 6e-81), which is induced in *M. sexta *fat body after bacterial challenge [73]. This *S. frugiperda *immulectin gene (Sf1H01505-5-1) seems to be preferentially transcribed in hemocytes (Table 2, H/F column) thus differing from other immulectin genes generally reported as synthesized in the fat body and secreted in the hemolymph [72]. Four immulectins are known in *M. sexta *[72], and we identified 3 different cDNAs in the *S. frugiperda *libraries. One of the other immulectins spotted in the microarray (Sf9L06812) is up-regulated by JcDNV in the fat body (+5.9), but is not modified by HdIV, suggesting that different immulectin genes may be differentially affected by HdIV. Down-regulation by HdIV of the *S. frugiperda *immulectin-2 homologue could be linked to inhibition of encapsulation observed in parasitized caterpillars. Interestingly, this gene is also down-regulated by JcDNV in the hemocytes (-3.4 in quantitative RT-PCR), suggesting an important immune function of this protein.

##### Calreticulin

In fat body, the highest fold change (-2.6 in microarrays and -3.0 in quantitative RT-PCR) was found for the transcripts related to the multifunctional Ca-binding protein calreticulin (CRT). Transcript levels for CRT also decrease in the hemocytes (-2.0 in microarrays and -2.9 in quantitative RT-PCR). Conversely, no variation in the number of transcripts was noted after injection of either JcDNV or heat-inactivated HdIV, suggesting that this variation is related to HdIV gene transcription. CRT is a conserved multifunctional Ca^2+^-binding protein, detected in a large variety of cellular compartments (reviewed in [74]). Intracellular CRT functions as a molecular chaperone and also regulates Ca^2+ ^homeostasis [75]. CRT is also found on the cell surface where it may play a role in cellular adhesion and migration [76] or clearance of apoptotic cells [77]. In insects, CRT has several functions, including functions associated with immunity. In *Galleria mellonella*, CRT protein was detected in the soluble fraction of hemocyte lysate and surrounding DEAE beads thus suggesting that CRT participates in the non-self recognition of early-stage encapsulation response [78]. The role of CRT in lepidopteran cellular response is also suggested by its presence on the surface of *Pieris rapae *hemocytes during phagocytosis of yeast cells [79]. Recently, a CRT was identified in the venom fluid of the parasitoid wasp *Cotesia rubecula *and shown to inhibit host hemocytes *in vitro *spreading [80]. The authors suggest that the wasp CRT functions as an antagonist of host CRT. In the *Hyposoter didymator *model, the host CRT gene is down-regulated in HdIV-infected larvae, suggesting a different strategy than *C. rubecula*. The down-regulation of CRT in HdIV-infected *S. frugiperda *larvae occurs in the same range in both hemocytes and fat body and may thus be responsible for the decrease in non-self recognition and encapsulation efficiency.

##### Scavenger receptor

One *S. frugiperda *gene that is down-regulated in response to HdIV injection (1.7/3.1-fold decrease for the two spots, Table 1) encodes a protein with significant similarities with *Drosophila *class C scavenger receptors (SR-C) (blast E value = 5e-46 with GenBank:AAW79423). The *S. frugiperda *gene encodes a predicted transmembrane protein that contains a MAM domain and two tandem complement control protein (CCP) domains. In *Drosophila*, the functionally characterized SR-C, dSR-CI, is implicated in phagocytosis and MAM and CCP domains are sufficient for binding of bacteria [81]. Scavenger receptors (SRs) are probably also involved in phagocytosis in Lepidoptera. Indeed, adhesion of *E. coli *on *Spodoptera littoralis *granular hemocytes is inhibited with polyinosinic acid, a specific ligand of SRs [82]. Since the dSR-CI recognizes a broad range of polyanionic ligands, similarly to the mammalian class A scavenger receptors [83], SR-C may be the SR responsible for the attachment of bacteria to lepidopteran hemocytes. To test the involvement of *S. frugiperda *SR-C in cellular responses other than phagocytosis, we analyzed the transcript levels of this gene after injection of large Sephadex beads. Beads are fully encapsulated 8 hours after injection and a preliminary macroarray analysis has indicated up-regulation of the SR-C gene 6 hours after injection (not detected 1 hour after injection, Volkoff, unpub.). So SR-C transcript levels were analyzed 6–7 hours after injection by quantitative RT-PCR in 2 different technical and biological conditions. Results indicate that SR-C is up-regulated (2.0/4.0-fold increase) in hemocytes after injection of Sephadex beads (Table 3), strongly suggesting that this novel SR-C plays a role in the *S. frugiperda *cellular response against parasites. The SR-C gene is down-regulated only when HdIV genes are transcribed (non significant with heat-inactivated virus and with JcDNV; Table 2). We thus found two opposite effects on SR-C gene transcript levels for the beads and the virus, suggesting that HdIV acts directly on SR-C transcript levels to impair the host cellular response.

##### Others

In addition to the three genes discussed above, HdIV injection down-regulates genes encoding proteins potentially involved in hemocytes adhesion and migration. For example, HdIV injection alters transcript levels of the annexin IX-B (decrease 1.6-fold), a calcium binding protein that plays a role in biological important functions such as membrane fusion and control of cell proliferation and differentiation [84]. In *Drosophila*, annexin IX is an immune responsive gene that is up-regulated in response to bacterial immune challenge [56,57]. Interestingly, a new discovered hemicentin-like gene (Volkoff *et al*., unpub.), a putative adhesion molecule of unknown function, is down-regulated in infected hemocytes (fold decrease -2.1/-2.2) as well as a transcript encoding a protein with significant similarity with the C-terminal region of the silkworm humoral lectin (hemocytin, -1.9), an adhesive protein related to encapsulation of foreign substances [85].

Polydnavirus, as other immunosuppressive virulence factors, commonly results in disruption of the hemocytes actin cytoskeleton [24]. Microarrays indicate that, besides down-regulating the two tubulin subunits in the hemocytes and in the fat body, injection of HdIV affects transcripts of several genes encoding proteins involved in actin-based motility processes, such as thymosin (-2.2), profilin (-1.85) and cofilin (-1.85). The reason for the down-regulation of structural cytoskeleton proteins (actin, alpha- and beta-tubulin) in the fat body is not known, and could be related to modifications in cellular trafficking, mitosis, or apoptosis (preparation to pupation). Actin-binding proteins are commonly found as up-regulated in genome-wide analyses of immune challenged *Drosophila *[8,57]. Inhibition of regulatory proteins of the actin cytoskeleton by HdIV is concordant with recent analyses in CsIV-infected hemocytes suggesting that actin is depolymerised or synthesized in lesser amounts [35].

Presence of HdIV also down-regulates genes that encode proteins involved in the assembly of the extracellular matrix, such as type IV collagens, components of basement membranes. Both collagen alpha-1 (2 spots) and collagen alpha-2 mRNAs were less abundant after injection of HdIV (-2.4/-3.7 and -2.2, respectively). Components of basement membrane are secreted by hemocytes (mainly granulocytes in Lepidoptera) and integrity of the basement membranes seems to play a central role in non-self recognition by hemocytes (reviewed in [2]). Decreased transcripts of collagen in HdIV-infected hemocytes could be related to a less efficient cellular response, although the mechanism remains unclear.

### Some *S. frugiperda *genes display increased transcript level in hemocytes 24 hours after HdIV injection

Among the 18 genes that are up-regulated in the hemocytes after HdIV injection, 11 encode proteins with similarities with known proteins (Table 1). The highest transcript levels variations were observed for the genes encoding galectin and the prophenoloxidase subunits 1 and 2 (PPO-1 and PPO-2).

#### Galectin

Two genes encoding sequences similar to galectins display an important increase of transcript levels 24 hours after HdIV injection in *S. frugiperda *hemocytes (average of 5.6-fold in microarrays and 10.0-fold in quantitative RT-PCR; Table 1). Galectins are thiol-dependent β-galactoside-binding lectins found in a large variety of organisms (reviewed in [86]). The *S. frugiperda *galectin gene is specifically transcribed in hemocytes (Barat-Houari, unpub. results) and encodes a protein containing a single carbohydrate recognition domain, which is more similar to the mammalian galectin-9 than to other described insect galectins. The *S. frugiperda *galectin gene is also up-regulated in hemocytes following JcDNV injection (6.5-fold increase), but down-regulated after injection of heat-inactivated HdIV (1.6-fold decrease; Table 2). In vertebrates, galectins are involved in a variety of cellular processes such as adhesion [87], apoptosis [88] and they possibly regulate innate immune responses [86]. In insects, galectins are thought to have dual functions in development and innate immunity (reviewed in [89]). In *Anopheles gambiae*, two galectin genes, *GALE8 *and *GALE5 *are induced by bacterial and malarial challenges [90]. In *Drosophila*, the Dmgal galectin is expressed in hemocytes and may participate in recognition of microorganisms or, alternatively, modulate hemocyte aggregation during infection [89]. It is possible that increased *galectin *levels in HdIV-infected *S. frugiperda *larvae might lead to the abnormal clumping of hemocytes observed in parasitized larvae (Volkoff, pers. data), thus contributing to a reduction in the number of circulating hemocytes capable of forming the capsule, and to a less efficient cellular response [91,92].

#### Prophenoloxidase subunits

The two *S. frugiperda *genes encoding the two prophenoloxidase subunits 1 and 2 (PPO-1 and PPO-2) showed increased transcript levels in microarray experiments. The microarray contains 4 cDNAs encoding the PPO-1 protein and 3 cDNAs encoding the PPO-2 protein and all of these cDNAs are significantly up-regulated (increases 1.9/4.9-fold and 4.0/5.9-fold, respectively; Table 1). This result was confirmed with quantitative RT-PCR on other biological samples (Table 2). The level of transcripts for the proPO-1 gene was still higher after injection of heat-inactivated HdIV (+7.6), suggesting that the up-regulation of this gene is not directly related to HdIV transcription, but could be linked to presence of HdIV in the caterpillar and thus could reflect a response to HdIV presence. The response seems specific to HdIV since, as shown in Table 2, the transcript level was not changed in caterpillars 24 hours after injection of the densovirus JcDNV. In *Drosophila*, none of the three proPO genes is induced after microbial infection [56] and enzyme activation does not seem necessary for resistance to microbial infections [93]. Conversely, in mosquito, the expression of two pro-POs is significantly enhanced in response to *microfilariae *inoculation and blood feeding, respectively [94]. An inducible proPO was identified recently in *Spodoptera litura*, and its transcript level increases after bacterial infection [95]. Thus the important up-regulation of the two *S. frugiperda *pro-PO genes by HdIV, inactivated or not, may indirectly reflect a response of this insect to enveloped viruses such as HdIV. This would corroborate previous reports on a possible role of PO in antiviral response in the lepidopteran larvae [16]. We cannot exclude that the highest level of proPO transcripts detected in *S. frugiperda *hemocytes could also be related to a selective destruction of a subset of hemocytes that are responsible of proPO enzyme production. Indeed, *Microplitis demolitor *bracovirus (MdBV) selectively induces apoptosis of the granulocytes [96]. However, no clear hemocyte apoptosis is observed after infection with HdIV, contrary to this hypothesis. In HdIV-injected *S. frugiperda *larvae, concomitant to increased *proPO *transcript levels, by 24 hours after HdIV injection, the *Sf_PPAE *transcript levels have decreased. This should result in a general decrease in PO activation and in the inhibition of melanisation observed in parasitized caterpillars.

## Conclusion

Polydnavirus or calyx fluid injection was previously reported to affect host protein levels, by inhibiting translation or interfering with post-transcriptional steps in the parasitized host. The transcriptomic analysis of *S.frugiperda *genes conducted here shows that injection of filter-purified HdIV results in modifications of transcript levels for several host genes. We cannot assert that all the variations detected by this microarray analysis result exclusively from HdIV, but filter purification is a method that minimizes contaminations with wasp proteins [97]. Moreover, results obtained for the genes studied after injection of heat-inactivated HdIV suggest that HdIV transcription is required for the changes observed. Whether the observed variations in host genes transcript levels result from a direct effect of HdIV proteins or from upstream regulations of other genes or proteins in major pathways will need to be further investigated. The fold-changes observed for transcript levels of the down-regulated genes are relatively low (-1.9 in average and maximum -3.7 for collagen in microarray experiment, -3.1 in average and maximum -4.5 for immulectin in quantitative RT-PCR assay), which is consistent with microarray analyses performed in other biological models and for which high fold changes are usually not observed. These results suggest that HdIV infection decreases transcript levels but does not shut-down a large panel of host genes. Moderate and diversified effects on global host physiology at the immune response level as well as at the developmental level are compatible with maintenance and survival of the host during all parasitoid development. Based on our knowledge on other insects, we can conclude that several of the steps in the lepidopteran host immune response are affected. Down-regulation of putative pattern recognition molecules such as calreticulin and immulectin suggests a decreased recognition efficiency of foreign bodies. Decreased levels of transcripts for lysozyme and PPAE indicate that the humoral response is affected. Finally, the cellular response seems to be impaired due to interference with transcript levels of cytoskeleton-related proteins (actin regulatory proteins, tubulins) or scavenger receptors. The next step will be to better characterize these regulated genes at a genomic level, in order to identify common signaling pathways.

This study also raises the question of the function of the proteins encoded by genes affected by the HdIV injection in lepidopteran immune defense and development. Our results suggest an important role for calreticulin, and two novel proteins, scavenger receptor and hemicentin-like proteins, in lepidopteran immune response, and makes lysozyme and PO good candidates as molecules involved in virus recognition and antiviral defense.

Based on the results obtained, we plan now to analyze the interaction between HdIV and the lepidopteran host *S. frugiperda *using a more complete microarray comprising a higher number of probes. This should circumvent the limits of the microarray analysed in this study, particularly the reduced number of genes that were screened. Analyzing the transcript levels of homologues of known components of major pathways involved in insect immune response such as Toll, Imd or Jak/Stat pathways and those of genes previously shown to play a role in the formation of the hemocytic capsule, such as the plasmatocyte spreading peptide cytokine [98,99], or integrins [19,100] or the immunoglobulin domains-containing hemolin (review in [5]), should provide an enhanced outline of the immune-suppressive effects of HdIV.

To conclude, this study, although based on a simplified biological model, provides evidence that infection with the polydnavirus HdIV affects, directly or indirectly, transcript levels of lepidopteran host genes. The results obtained in the present study also validate the transcriptomic approach to analyze the complex interactions in the parasitoid-polydnavirus-host models. Further use of such global approaches should result in a better understanding of the strategies employed by parasites to manipulate their host physiology, and should give new insights on lepidopteran innate immunity by allowing the identification of potential targets of the immunosuppressive polydnaviruses.

## Methods

### I/Array design description: construction of *Spodoptera frugiperda *cDNAs microarrays

The microarrays were glutaraldehyde activated aminosilane-coated glass slides (pre-cleaned gold seal microslides, Merck) on which we spotted in triplicates PCR products corresponding to *S. frugiperda *cDNAs. We used 1750 non-redundant sequences from 4 *S. frugiperda *cDNA libraries (1266 cDNAs from a Sf9 cell line library, 381 from a hemocyte library, 48 from a fat-body library and 55 from a midgut library; sequences available at [61]; [101]) and 11 viral cDNAs as a control.

The DNA probes were amplified by PCR using primers flanking the cDNA inserts, the forward primer 5'-GTGTTGGTACCCGGGAATTCG-3' and the reverse primer 5'-GCTCGAGTCTAGAGTCGACTG-3'. These primers had a C6 amine modification in the 5' end in order to bind to the glutaraldehyde activated aminosilane-coated slides. After amplification, the quality of the PCR products was verified by electrophoresis on 0.8% agarose gels and the DNA purified using QIAquick 96 PCR purification kit (QIAGEN) according to manufacturers' protocol. After quantification using PicoGreen (Molecular Probes), all PCR products were transferred to 384-well plates, lyophilized and re-suspended in 3 × SSC to a final concentration of 0.150 mg/ml. The spotting was done with a Chip WriterPro spotter (Virtek, Biorad) using PT3000 pins supplied by EBN (Belgium). Each pin withdraws a volume of about 250 nanoliters and deposits a spot volume of about 0.6 nanoliters, with a diameter of approximately 90 to 130 μm. The cDNAs were printed on the slides using an element center-center spacing of 180 μm. After printing, slides were allowed to dry and then used immediately, or were stored desiccated at room temperature. Slides exhibiting defects such as missing or damaged spots were discarded.

Before hybridization, slides were post-processed in several steps as described in Hugot *et al. *[102] : (a) Unbound DNA was removed by washing with 0.2% (w/v) SDS and double-distilled water, (b) covalently bound DNA was denatured for 2 min in boiling water, (c) free aldehydes were reduced by soaking slides for 5 min in 68 mM sodium borohydride (dissolved in PBS containing 25% (v/v) ethanol), and (d) free glasses sites were blocked by an incubation of 30 min at 60°C in the presence of 0.2% (w/v) casein. Several washing steps were performed with 0.2% (w/v) SDS and double-distilled water. After washes, the post-processed slides were dried by centrifugation at 500 g for 5 minutes and then either used immediately or stored desiccated at room temperature.

### II/Microarray experiment description

#### 1- Experimental design

We performed 2 biological assays, the first with the polydnavirus HdIV and the second with the densovirus JcDNV. Each assay was composed from two sets, named "infected" and "non-infected control", and each set was composed of 20 1-day old *S. frugiperda *last instar larvae. For each pair infected/non-infected samples, a second labeling reaction, named dye-swap, was performed with inversion of the fluorescent dyes. For each assay, we prepared 3 independent biological replicates and 2 dye swap per biological replicate, i.e. 6 slides per assay.

#### 2- Sample used, extract preparation and labelling

##### Virus preparation and insect infections

*Hyposoter didymator *and *S. frugiperda *were reared as previously described in Volkoff *et al. *[29]. HdIV suspension was prepared as previously described [32]. Briefly, ovaries from 40 *H. didymator *females were dissected and dislocated in phosphate-buffered saline (PBS) and then filtrated with a 0.45 μm acetate-cellulose filter. The HdIV suspension obtained was adjusted to 600 μl and then each *S. frugiperda *larva was injected with 27 μl (corresponding to 1.8 ovaries/larvae). Each larva from both HdIV-infected and control sets were injected with 27 μl of either HdIV suspension or PBS.

We used the densovirus isolated from the lepidopteran *Junonia coenia *JcDNV [103]. The densoviruses belong to *Parvoviridae *family and produce highly contagious lethal diseases in invertebrates [62], and *Spodoptera *species are particularly sensitive to JcDNV. The control and viral inocula were prepared by crushing either healthy or JcDNV-infected *S. frugiperda *in 1.5 ml of PBS containing 2% ascorbic acid, pH 7.2. The homogenates were clarified 10 min at 9000 g, filtrated on a 0.45 μm acetate-cellulose filter, and then diluted ten fold. Each larva from the JcDNV infected or control sets were inoculated with 13 μl of healthy or viral suspension.

##### RNA extraction from *S. frugiperda *tissue samples

Samples were collected 24 hours post-injection (24 h p.i.) from both virus-infected and non-infected control sets. Total RNA was extracted from tissue samples (hemocytes or fat body cells) using the Rneasy Mini Kit Total RNA according to manufacturers' protocol (Qiagen S.A., Courtaboeuf, France, cat. no. 74106). Hemocytes were collected as previously reported[32]. Dissected fat body was collected directly in the extraction buffer (RLT buffer) from the Rneasy Mini Kit. On-column DNase digestion was then performed with the RNase-free DNase Kit (Qiagen, cat. no. 79254). Additionally, for the JcDNV-infected samples, infection was controlled by RT-PCR using an oligodT primer and a primer specific to the JcDNV *viral protein *gene.

##### RT and labeling

Ten μg of total RNA were labeled with either Cy5dCTP or Cy3dCTP using the ChipShot Labeling System (Pronto!™ Plus System, cat. no. 40054-55, Promega; [104]), according to manufacturers' protocol. We used the dyes from Amersham Biosciences. Non-incorporated dyes were eliminated using the ChipShot Labeling Clean-Up System (Pronto!™ Plus System, cat. no. 40054-55, Promega). After quantification of each dye incorporation, the labeled cDNAs were dried in speed-vacuum and then dissolved in the required volume of water-diluted Dig Easy hybridization solution (1/3 v/v)(DIG Easy Hyb Granules, Roche Diagnostics GmbH cat. no. 1 796 895).

#### 3- Hybridization procedures and parameters

The labeled cDNAs obtained from the two experimental conditions (i.e. infected and non-infected samples) were mixed in equivalent quantities (pmols) of incorporated Cy3 and Cy5 in order to obtain a final volume of 20 μl hybridization solution. This solution was then put on the array surface and a micro cover glass (22 × 32 mm) was slowly applied to allow the solution spreading and covering the whole spotted area. Slide hybridization was done in Corning CMT-hybridization chambers overnight by total immersion in a water bath at 42°C, in the dark.

Following overnight hybridization, the slides were washed according to standard procedures [102] and dried by centrifugation at 500 g for 5 min before scanning.

#### 4- Data measurement and specifications of data processing

##### Image analysis and quantification

Hybridized slides were scanned with an Axon 4000 B microarray scanner (Axon Instruments, Foster City, CA) to generate 16-bit TIF images. The laser power was set at 100% and the photo-multiplier tube (PMT) gain for each wavelength between 600 and 900. Images were analyzed with GenePix Pro4.0 Axon software (adaptative circle segmentation algorithm), according to manufacturers' instructions.

##### Data standardization and normalization

After microarray raw data acquisition, data processing was performed using the web-accessible MicroArray Data Suites of Computed Analysis, MADSCAN resource (at [105]; [106]). The MADSCAN procedure uses the median intensities after background corrections and analytical parameters provided by GenePix Pro. MADSCAN perform physical validation and quality filtration based on five-step criterion. During these first steps, several spots were ruled out from the analysis. It was the case for viral controls (one JcDNV and HdIV positive controls: rep1, rep2) that displayed poor quality spots and were flagged by the different quality control steps (Genepix or MADSCAN). After data filtration, MADSCAN perform log transformation of the median intensities to makes the distribution of the data symmetrical and almost normal. Hence, the scaled lowess fitness (LOcally-WEighted Regression) normalization algorithm was applied to minimize signal-dependent non-linear bias between the two intensity levels. Madscan perform a within-print tip (local) normalization with a smooth parameter (defined as the fraction of data used to smooth at each data point) *f *= 0.40 [107].

#### 5- Microarray statistical analysis

To identify differentially expressed genes, the lowess normalized values of the background corrected intensities obtained from the red and the green channels calculated by MADSCAN were then implemented in two validated statistical analysis programs: the Significant Microarray Analysis (SAM) downloaded from [108] and the GeneAnova program [109]. Only the data significant with the two statistical methods were considered.

For the SAM analysis we kept all the genes with enough non-missing values as program is able to input missed values with the average of the 10 non-missing nearest-neighbor among replicates. We applied the two-class unpaired method from the SAM package to test the null hypothesis of no effect of viral compared to control injection across 18 replicates (3 biological × 2 technical dye-swap × 3 within slide as each gene is present in triplicate). This method is a nonparametric test that call significant genes based on the comparison of the experimental score d(i) to its expected value calculated from N random permutations between treated and untreated sample data (random assignment of treatment). Then, from the randomized data, the SAM program calculates the ratio of the number of false-positives to the expected number of genes called significant. This value provides an estimate of the number of genes identified by chance, the False Discovery Rate (FDR). The *q-*value, calculated from the distribution of the d(i) statistics obtained from permutations, represents the lowest FDR at which the gene is called significant. We selected differentially expressed genes that met both FDR criteria (estimated FDR median and 90th percentiles of 0%) and fold change criteria (at least 1.5-fold) [110]). The 1.5 threshold was chosen according to Yang *et al. *[111], who suggest that fold changes smaller than ± 2 can reliably identify differentially expressed genes when sufficient number of replicates and flip-dye assays are performed. Afterwards, the results were ascertained with an ANOVA based microarray analysis, GeneANOVA, which is a freely available gene expression analysis of variance software developed by The Laboratory of "Génome et Informatique" at Evry-Génopole [109]. As GeneANOVA requires no missing values, we implemented the program with the SAM imputed data set in which the missed values has been calculated from the mean of 10 non-missing nearest neighbour. For this analysis we constructed a statistical model including 5 factors: genes, type of injection (HdIV versus control or JcDNV versus control), dye label, technical dye-swap replicates and biological replicates. Theses factors represent a set of interacting parameters reflecting differential gene expression across different experimental conditions. ANOVA model allows an estimation of the contribution of each factor from the study design in the total variation of the whole set of measurements and the pair-wise sets of interaction between factors: gene-by-condition, gene-by-slide (i.e. dye-swap technical replicate), gene-by-biological replicate. GeneANOVA gives also the significance of each contribution globally and for each gene. When assessing genes-by-injection interaction, transcriptionally modulated genes identified from the SAM analysis, presented a significant interaction with injection. ANOVA analysis revealed no effect of confounding factors such as dye label and replicates on gene expression levels (i.e. no significant interaction between those factors and injection p > 0.01), suggesting that the transcriptional changes detected by our microarray experiment are independent from dye labeling, technical replicates and biological samples.

### III- Quantitative RT-PCR

The genes retained for real-time quantitative RT-PCR additional investigation, calreticulin, lysozyme, immulectin, the two prophenoloxidases, galectin, scavenger receptor and proPO-activating enzyme, were chosen because of their high fold-change following HdIV infection and their involvement in different immune response mechanisms. All genes were analyzed in hemocytes, a tissue in which all the selected genes, with the exception of calreticulin, are preferentially transcribed (illustrated by the ratio H/F, Table 2). In the fat body, we restricted analysis for JcDNV-regulated gene (lysozyme) and for calreticulin gene, the main fat body HdIV target.

#### 1- Sample preparation

For real-time quantitative RT-PCR experiments, we repeated the same biological design than what previously described for micro-array assays; a fourth biological replicate was used for control larvae (injected with PBS) in the HdIV assay. Two additional assays were included: an immune challenge assay, and an HdIV-heat inactivation assay.

##### Immune challenge by injection of beads

The immune challenge assay consisted in injection of Sephadex beads of 20–50 μm diameter (Sigma G-25) into last instars *S. frugiperda *using an 18 G needle. For kinetics analyses by transmission electron microscopy (TEM), we used latex beads (Sigma SD-91; 90 μm diameter). Both latex and Sephadex beads induced an encapsulation response in *S. frugiperda *larvae. For TEM observations, larvae were dissected in PBS at different times after injection (30 min, 1, 2, 3, 4, 6, 8 and 15 hours) and the beads were individually recovered. For RNA purification, hemolymph containing total hemocytes (circulating hemocytes as well as encapsulated beads) was collected 6 hours or 7 hours after injection.

##### Inactivation of HdIV

The aim of the inactivation was to have virus particles susceptible of being recognized by the caterpillar, but no transcription of viral genes. Absence of virus transcription was controlled by RT-PCR using specific primers of the viral innexin-1 gene [48]. Since UV-inactivation did not result in the complete elimination of HdIV transcripts (data not shown), the viral suspension was treated 10 minutes at 95°C. Integrity of the heat-inactivate virus particles was controlled by negative staining. To verify that heat-inactivate virus particles were still able to infect host tissue, hemocytes collected from 5 injected larvae were observed by TEM. We found a smaller overall amount of virus in the samples, but still found particles within the hemocytes (Figure 2).

#### 2- Transmission electron microscopy (TEM)

Preparation for TEM observations was identical for hemocytes collected from larvae injected with beads or with HdIV (inactivated or not) and for pelleted encapsulated beads. Cells were pelleted by a gentle centrifugation, washed three times in PBS and fixed with 2% (v/v) glutaraldehyde in 0.1 M cacodylate buffer pH = 7.4 for overnight at +4°C then post-fixed with 2% (v/v) osmium tetroxide in the same buffer for 1 hour at room temperature. Samples were dehydrated through an ethanol series and embedded in Epon. Ultrathin sections contrasted with uranyl acetate and lead citrate were examined under an electron microscope Zeiss EM 10 CR at 80 KV.

#### 3- RNA preparation and reverse-transcription

RNA samples were prepared using Rneasy Mini Kit Total RNA as described above for micro-array assays except an additional step to fully remove cellular and viral genomic DNA. To do this, 8 μg of total RNA was incubated at 37°C for 3 hours with 8 units Rnase-free RQ1 DNAse (Promega). RNA samples were then ethanol precipitated with sodium acetate, washed twice in 75% ethanol and re-suspended in 8 μl of nuclease free water. We controlled the absence of genomic DNA from both cellular and viral origin by subjecting genomic DNA free RNA to two PCRs (1 μg per amplification), firstly to amplify a cellular gene, ELF-1, and secondly a viral specific viral innexin-1 gene. Thus, the 6 μg remaining genomic DNA-free total RNA, was reverse transcribed into cDNA using SuperscriptII according to manufacturers' protocol (final volume reaction of 20 μl), to obtain a 0.3 μg/μl theoretical concentration of cDNA.

#### 4- Quantitative RT-PCR

Primer pairs for quantitative RT-PCR for 8 selected genes and 3 endogenous reference genes (Ubiquitin E2, RNA polymerase II and ATP synthase; sequences available in SpodoBase; URL: [61]) were designed using the software package Primer Express™ from Applied Biosystems, according to the default parameters optimized for the ABI Prism 7000 Sequence Detection System (SDS). Primer sequences and amplicon sizes are available in Additional file 3. For each sample, quantification of gene transcript levels was performed twice in 96-well PCR plates (ABgene). Two different plate designs were obtained by permutation of the plate positions between the whole set of infected and the whole set of non-infected samples. Both plate design comprise triplicate of each biological replicate and were used in two independent quantitative PCR runs. Hence, as for the microarray assays, we analyzed data from a total of 18 replicates at least (3 biological replicates × 3 within plate technical replicates × 2 technical replicates i.e. repeated measurements) obtained from each real time PCR experiment. The PCR was conducted in a 25 μl final volume reaction with a PCR mix containing 5 μl of cDNA at 4 ng/ul (i.e. a quantity of cDNA corresponding to 20 ng of total RNA), 1 X PCR buffer (Invitrogen), 3 mM of MgCl2, 200 μM dNTP mix (Invitrogen), 0.2 μl of 1/2000 dilution stock solution of SYBR green I (Invitrogen), 0.5 μM of ROX dye (Interchim), 0.4 μM of primer pairs and 0.1 U of Platinium Taq DNA polymerase (Invitrogen).

We used the Applied Biosystem 7000 sequence detection system according to the recommended amplification scheme: 95°C 2 min and 40 cycles: 95°C 15 sec, 60°C 1 min. The dissociation curve method was applied according to the manufacturer's protocol (60°C to 95°C) to unsure the presence of a single specific PCR product.

#### 5- Quantitative data analysis

As other methods for quantitative RT-PCR data analysis are based on the assumption that, for a given amplicon, the PCR efficiency among all individual are similar, we preferred to analyze our data with an assumption-free alternative method. We used the LinReg PCR program developed by Ramakers and coll. [112] that uses the fluorescence of each sample per cycle. This program calculates the starting concentrations of mRNA, which correspond to the intercept of the curve and the slope corresponding to the PCR efficiencies of each individual sample. This analysis uses the Rn values (normalized reporter), which are the fluorescence emission intensity of the reporter dye (SYBRGreen) divided by the Rox passive reference dye intensity. This approach gives the initial number, named N0 value, of molecules presents in each sample. Thus, for each of the 8 genes of interest and the 3 endogenous reference genes, the arithmetic mean of N0 value was calculated from 6 technical replicates for the 3 or 4 biological replicates. Then, for each tissue and each injection type, we calculated a normalization factor (NF) as described by Vandesompele *et al. *[113], from the geometric mean of the N0 arithmetic mean obtained for each of the 3 endogenous reference genes from all replicates. The N0 arithmetic means from each biological replicate were divided by the corresponding NF (tissue injection) to obtain the relative N0 values corresponding to the initial transcript level of each gene in a given tissue and condition. Then, normalized N0 means were compared between infected and non-infected samples using the SAM program as for microarrays analyses and fold changes were calculated from the quantitative RT-PCR measurements.

## Abbreviations

AMP: antimicrobial peptide

CRT: Calreticulin

HdIV: *Hyposoter didymator *ichnovirus

JcDNV: *Junonia cœnia *densonucleosis virus

MAM: Meprin, A5 antigen and receptor protein tyrosine phosphatase Mu domain

PO: phenoloxydase

PPAE: prophenoloxidase activating enzyme

SR-C: class C scavenger receptor

TEM: Transmission Electron Microscopy

## Authors' contributions

The study was conducted by M. Barat-Houari, F. Hilliou, F.X. Jousset and A.N. Volkoff, with the help of J. Rocher and L. Galibert; statistical analyses were performed by M. Barat-Houari; M. Ravallec was in charge of electron microscopy preparations; microarrays were spotted by L. Sofer; bioinformatics analysis for microarray construction were conducted by E. Deleury; P. Delobel participated in the scanning and interpretation of microarray results; R. Feyereisen and P. Fournier participated in the study design.

## Supplementary Material

Additional file 1 Complementary results of microarray analysis.**1A: **Results obtained for the HdIV cDNAs spotted on the microarray. Results obtained for the HdIV cDNAs spotted on the microarray, in the hemocytes (H) and in the fat body (FB) 24 hours after injection of HdIV, as detected by a modified t-test from the SAM package and an ANOVA based microarray analysis, GeneANOVA. Only the genes with a fold change superior or equal to 1.5 with both a "false discovery rate" (FDR) median and 90^th ^percentiles of 0% were kept. The SAM fold and q-value (the lowest FDR at which the gene is called significant) as well as the ANOVA F "gene-condition" value and the p-value are given. Gene Name: annotation of the sequence. (B) List of additional clones corresponding to each gene. List of additional cDNA clones that have been spotted on the microarray and which corresponded to the same gene. Click here for file

Additional file 2 Functional *Spodoptera frugiperda *gene classification. Functional gene classification based on the one described in Shida *et al. *for the ascidian *Ciona intestinalis *[114], and used for the annotation of the *Spodoptera frugiperda *sequences (Volkoff *et al.*, in prep.). Click here for file

Additional file 3 List of the primers designed for quantitative RT-PCR analysis. List of the forward and reverse primers designed for quantitative RT-PCR analysis of the 8 selected genes and the 3 endogenous reference genes. Click here for file

## Figures and Tables

**Table 1:** List of *Spodoptera frugiperda *genes transcriptionally modified by HdIV infection.

**Spodobase ID**	**Gene Name**	**Functional class**	Number of clones				**SAM Fold**	**GeneANOVA**	
			H	F	M	L		F gene-cond°	p-value
**Hemocytes_HdIV : 72 significant genes, 18 up-regulated and 54 down-regulated**							**q-value 0.0056**		

Sf1H03393-5-1	Fumarate hydratase	AVI tricarboxylic acid cycle	1	3	0	0	**2.24**	74	<0.00001
Sf1H00151-5-1	retinol dehydratase; iso-1	AVI catalytic	7	0	0	0	**2.11**	152	<0.00001
Sf9L04641	90-kDa heat shock protein HSP83	AVII stress. hsp	0	0	2	5	**2.19**	33	<0.0001
Sf1H03026-5-1	heat shock cognate 70 protein	AVII stress. hsp	1	0	0	0	**1.58**	62	<0.00001
Sf1H00903-5-1	prefoldin subunit 2	AVIII processing	2	0	1	0	**1.88**	91	<0.00001
Sf1H00522-5-1	Galectin; iso-1	BIII lectin	3	0	0	0	**5.66**	161	<0.00001
Sf1H01648-5-1	Galectin; iso-2	BIII lectin	2	0	0	0	**5.50**	110	<0.00001
Sf1H01841-5-1	lectin (CrV)	BIII lectin	11	0	0	0	**1.66**	44	<0.00001
Sf1H03263-5-1	MBF2	C regulator	10	3	0	0	**2.18**	NS	<0.00001
Sf1H00908-5-1	PPO-1 (X 4 spots)	DI PPO	75	0	0	0	**[1.9-4.89]**	[27–144]	<0.00001
Sf1H00508-5-1	PPO-2 (X 3 spots)	DI PPO	60	0	0	0	**[4.02–5.91]**	[67–104]	<0.00001
Sf1H01637-5-1	Hypothetical protein A.gambiae	EII	1	0	0	0	**2.73**	250	<0.00001
Sf9L08591	Hypothetical protein Drosophila	EII	0	0	1	1	**2.52**	96	<0.00001
Sf1H00837-3-1	No Similarity	EIII	1	0	0	0	**2.74**	134	<0.00001
Sf1H00871-5-1	No Similarity	EIII	1	0	0	0	**2.98**	261	<0.00001
Sf1H01434-3-1	No Similarity	EIII	1	0	0	0	**3.03**	102	<0.00001
Sf1H02673-5-1	No Similarity	EIII	1	0	0	0	**1.96**	173	<0.00001
Sf1H00201-3-1	NS. similar to Hypothetical protein (5' seq)	EIII	2	0	0	0	**2.73**	93	<0.00001

Sf1H02439-5-1	Annexin IX-B	AI Ca binding	7	3	0	0	**0.62 (-1.61)**	66	<0.00001
Sf9L07393	Calmodulin	AI Ca binding	5	2	11	6	**0.59 (-1.69)**	31	<0.0001
Sf9L07135	Cytochrome C oxydase I	AI electron transport	162	117	232	146	**0.62 (-1.61)**	30	<0.0001
Sf1H02665-5-1	splicing factor 3a	AII splicing	1	0	0	0	**0.48 (-2.08)**	196	<0.00001
Sf9L06869	cofilin	AIV actin	16	1	10	2	**0.54 (-1.85)**	62	<0.00001
Sf1H03446-5-1	profiling	AIV actin	30	2	16	0	**0.54 (-1.85)**	142	<0.00001
Sf9L03392	Thymosin beta	AIV actin	7	1	3	13	**0.45 (-2.22)**	33	<0.0001
Sf9L06055	alpha tubulin	AIV tubulin	5	5	7	7	**0.56 (-1.80)**	56	<0.00001
Sf9L01568	beta-1 tubulin	AIV tubulin	1	2	2	1	**0.56 (-1.80)**	NS	<0.00001
Sf1H02495-5-1	synaptotagmin-like; granuphilin	AIX exocytosis	2	0	0	0	**0.65 (-1.54)**	27	<0.0001
Sf9L01210	Ribosomal protein S24	AV ribosomal	1	0	4	21	**0.58 (-1.71)**	84	<0.00001
Sf1H02303-5-1	kynurenine aminotransferase	AVI biosynthesis	2	0	0	0	**0.60 (-1.66)**	73	<0.00001
Sf1H01920-5-1	beta glucosidase; iso-1	AVI carbohydrate metabolism	1	1	0	0	**0.58 (-1.72)**	123	<0.00001
Sf1H01659-5-1	putative carboxylesterase	AVI catalytic	5	1	0	0	**0.56 (-1.78)**	87	<0.00001
Sf9L07277	enolase	AVI glycolysis	4	1	1	2	**0.55 (-1.82)**	NS	<0.00001
Sf9L06272	glyceraldehyde-3-phosphate dehydrogenase	AVI glycolysis	1	3	8	3	**0.63 (-1.58)**	20.82	<0.0001
Sf9L02512	fructose 1 6-bisphosphate aldolase	AVI glycolysis	0	0	0	1	**0.64 (-1.56)**	64.61	<0.00001
Sf9L02319	Deoxyribose-phosphate aldolase-like	AVI nucleotide metabolism	0	0	0	2	**0.64 (-1.56)**	72	<0.00001
Sf1H00106-5-1	Scavenger receptor;iso-1 (X 2 spots)	BI receptor	7	0	0	0	**[0.32–0.59] [-1.69/-3.11]**	[78–159]	<0.00001
Sf9L03085	Cyclophilin 5/peptidyl-propyl isomerase 5	BII PPIase	5	4	4	3	**0.61 (-1.64)**	34	<0.00001
Sf9L02900	similar to RAS suppressor protein 1	BII Ras signal transduction	0	0	0	2	**0.66 (-1.52)**	56	<0.00001
Sf1H01096-5-1	hemicentin (X 3 spots)	BIII CAM	11	0	0	0	**[0.45–0.48] [-2.10/-2.22]**	[117–132]*	<0.00001
Sf9L07414	collagen alpha 1 (IV) (X 2 spots)	BIII ECM	0	0	0	1	**[0.27–0.41] [-2.44/-3.70]**	[63–207]	<0.00001
Sf1H01457-5-1	collagen alpha 2 (IV)	BIII ECM	1	2	0	0	**0.45 (-2.22)**	203	<0.00001
Sf9L06099	cobatoxin-like protein; iso-1	DI AMP	0	0	0	2	**0.50 (-1.98)**	43	<0.00001
Sf1H01136-5-1	lysozyme (X 2 spots)	DI AMP	140	86	18	0	**0.46 (-2.17)**	114	<0.00001
Sf1H00867-5-1	hemocytin precursor	DI lectin	3	0	0	0	**0.52 (-1.93)**	111	<0.00001
Sf1H01505-5-1	immulectin-2	DI LPS binding	5	0	0	0	**0.52 (-1.93)**	99	<0.00001
Sf9L00974	calreticulin	DI multi-function	5	1	0	1	**0.51 (-1.96)**	38	<0.00001
Sf1H00566-5-1	PPO activating Enzyme	DI PPAE	4	0	0	0	**0.45 (-2.22)**	196	<0.00001
Sf1H02709-3-1	Hypothetical protein A. gambiae (X 5 spots)	EII	38	0	0	0	**[0.44–0.55] [-1.82/-2.27]**	[88–140]*	<0.00001
Sf1H02044-5-1	Hypothetical protein Drosophila	EII	1	0	0	0	**0.61 (-1.64)**	137	<0.00001
Sf9L08287	Hypothetical protein A. gambiae	EII	0	0	0	1	**0.50 (-1.99)**	47	<0.00001
Sf9L06591	Hypothetical protein Drosophila	EII	0	0	0	1	**0.57 (-1.75)**	36	<0.00001
Sf9L06826	Hypothetical protein Drosophila	EII	0	0	0	1	**0.66 (-1.52)**	26	<0.0001
Sf9L00177	similar to calponin; iso-1	EII	4	0	0	7	**0.59 (-1.69)**	31	<0.0001
Sf1H00681-5-1	similar to LPS-induced TNF-alpha factor (X 2 spots)	EII	15	2	0	1	**[0.47–0.52] [-1.92/-2.13]**	[123–159]	<0.00001
Sf1H03038-5-1	similar to pancreatic triacylglyceride lipase (X 2 spots)	EII	6	0	0	0	**[0.45–0.46] [-2.17/-2.22]**	[81–150]	<0.00001
Sf1H00115-5-1	similar to protease inhibitor (AC protein); iso-1	EII	3	0	0	0	**0.50 (-2.01)**	131	<0.00001
Sf9L04444	similar to Swiprosin	EII	3	0	0	1	**0.63 (-1.58)**	25	<0.0001
Sf1H00035-3-1	No Similarity	EIII	19	0	0	0	**0.31 (-3.19)**	88	<0.00001
Sf1H00499-5-1	No Similarity	EIII	11	0	0	0	**0.60 (-1.66)**	14	<0.001
Sf1H00686-5-1	No Similarity	EIII	10	0	0	0	**0.43 (-2.33)**	105	<0.00001
Sf1H03014-5-1	No Similarity	EIII	1	2	0	0	**0.64 (-1.56)**	178	<0.00001
Sf1F00357-5-1	No Similarity	EIII	1	0	0	0	**0.59 (-1.68)**	21	<0.0001
Sf1H00037-3-1	No Similarity	EIII	1	0	0	0	**0.56 (-1.78)**	212	<0.00001
Sf1H00879-5-1	No Similarity	EIII	1	0	0	0	**0.53 (-1.89)**	147	<0.00001
Sf1H01542-3-1	No Similarity	EIII	1	0	0	0	**0.43 (-2.33)**	348	<0.00001
Sf1H01712-5-1	No Similarity	EIII	1	0	0	0	**0.43 (-2.33)**	106	<0.00001
Sf1H02105-5-1	No Similarity	EIII	1	0	0	0	**0.41 (-2.44)**	163	<0.00001
Sf9L00090	No Similarity	EIII	1	0	0	4	**0.62 (-1.61)**	35	<0.00001
Sf9L01158	No Similarity	EIII	0	0	1	2	**0.45 (-2.22)**	73	<0.00001
Sf9L06863	No Similarity	EIII	0	0	0	1	**0.53 (-1.89)**	43	<0.00001
Sf9L08779	No Similarity	EIII	0	0	0	1	**0.52 (-1.91)**	NS	<0.00001

**Fat Body_HdIV : 7 significant genes, down-regulated**							**q-value 0.077**		

Sf9L07198	cytoplasmic actin A3	AIV actin	27	6	47	2	**0.59 (-1.69)**	27	0.00001
Sf9L06055	alpha tubulin	AIV tubulin	5	5	7	7	**0.45 (-2.22)**	135	<0.00001
Sf9L01568	beta-1 tubulin	AIV tubulin	1	2	2	1	**0.65 (-1.54)**	80	<0.00001
Sf9L07277	enolase	AVI glycolysis	4	1	1	2	**0.54 (-1.85)**	39	<0.00001
Sf9L02319	Deoxyribose-phosphate aldolase-like	AVI nucleotide metabolism	0	0	0	2	**0.51 (-1.96)**	82	<0.00001
Sf9L00974	calreticulin	DI multi-function	5	1	0	1	**0.38 (-2.63)**	85	<0.00001
Sf1F00299-5-1	Hemolymph glycoprotein precursor	DIII unknown	1	17	1	0	**0.51 (-1.96)**	33	<0.00001

**Fat Body_JcDNV : 8 significant genes, up-regulated**							**q-value 0.056**		

Sf9L00253	mRNA export factor binding	AII transcription coactivator	0	0	0	2	**1.87**	35	<0.000001
Sf9L07586	Ribosomal protein S27A	AV ribosomal	5	3	8	11	**3.82**	136	<0.000001
Sf1H03263-5-1	MBF2	C regulator	10	3	0	0	**1.54**	22	0.00006
Sf9L06099	cobatoxin-like protein; iso-1	DI AMP	0	0	0	2	**2.12**	70	<0.000001
Sf1H00171-5-1	lysozyme (X 2 spots)	DI AMP	140	86	18	0	**[1.59–1.66]**	[10–20]	0.0001
Sf9L06812	immunolectin-A precursor	DI LPS binding	0	1	0	2	**5.88**	137	<0.000001
Sf9L02793	No Similarity	EIII	0	0	0	1	**5.46**	212	<0.000001
Sf9L03186	No Similarity	EIII	2	1	7	21	**5.42**	149	<0.000001

List of *Spodoptera frugiperda *genes for which transcript levels are significantly modified in the hemocytes and in the fat body 24 hours after injection of HdIV, and in the fat body 24 hours after injection of JcDNV, as detected by a modified t-test from the SAM package and an ANOVA based microarray analysis, GeneANOVA. Only the genes with a fold change superior or equal to 1.5 with both a FDR median and 90^th ^percentiles of 0% were kept. For each assay, the SAM q-value (the lowest FDR at which the gene is called significant) is given. For each gene, the F value for "gene-condition" and the p-value calculated by GeneANOVA are given. Single genes for which more than one cDNA was spotted on the glass slide are indicated (x spots), in those cases, only the spot with the best score was kept in the list. Genes are organized according to their functional annotation (see Additional file 2 for legends). Spodobase ID: identity of the arrayed cDNA in the database Spodobase ([61]); Gene Name: annotation of the sequence after BlastX search against the GenBank nr database; Functional class: classes and subclasses to which the sequences belong to, based on the classification described in Shida *et al. *[114] for the ascidian *Ciona intestinalis *(details in Additional file 2); Number of clones: distribution of the cDNA clones corresponding to the given gene with available sequences in the different *S. frugiperda *cDNA libraries (H:hemocytes; F: fat body; M:midgut; L:Sf9 cell line). For genes represented by at least two cDNAs, we reported the interval [min, max] for the SAM fold change and for the F gene-condition from GeneANOVA analysis. Asterisk (*) indicate that one of the spots was non significant for GeneANOVA (clusters Sf1H01096-5-1 and Sf1H02709-3-1). The list of additional clones corresponding to each gene is provided in Additional file 1B.

**Table 2:** Variation in gene transcript levels in response to virus infection measured by quantitative RT-PCR.

		**Micro-array**		**RT-qPCR**																	
		
		**Fold (V/C)**		mean N0									**Fold (V/C)**		mean N0						**Fold (V/C)**
	**H/F**	HdIV	DNV	control PBS (n = 24)			HdIV (n = 18)			HdIV95° (n = 18)			HdIV	HdIV95	control Sf (n = 24)			DNV (n = 18)			DNV
	fold	fold	fold	min	**mean**	max	min	**mean**	max	min	**mean**	max	fold	fold	min	**mean**	max	min	**mean**	max	fold
**HEMOCYTES**																					

**PPO-1**	NS	**+4.9**	ND	0,73	**0,9**	1,1	11.7	**30.1**	48.5	3.8	**7.1**	10.5	**+32.0**	**+7.6**	3.5	**4.7**	5.9	4.5	**4.7**	4.9	NS
**PPO-2**	10.7	**+5.9**	ND	5.3	**6.1**	7.0	8.7	**43.8**	78.9	7.1	**7.9**	8.7	**+9.8**	NS	20.5	**24.3**	28.1	23.7	**27.0**	30.3	NS
**Galectin**	NS	**+5.6**	ND	0.7	**0.9**	1.1	3.8	**8.9**	14.1	0.3	**0.5**	0.7	**+10.0**	**-1.7**	0.6	**0.8**	0.9	4.0	**4.8**	5.6	**+6.5**
**Scavenger R**	26,0	**-3.1**	ND	10,6	**11,8**	13,0	1,8	**4,3**	7,0	8,4	**9,5**	10,6	**-2.8**	NS	22.0	**25.0**	27.9	21.5	**24.2**	26.9	NS
**PPAE**	26.7	**-2.2**	ND	7,0	**9,1**	11,2	1,7	**4,3**	7,0	6.5	**7.2**	7.8	**-2.11**	NS	7.3	**7.9**	8.5	9.2	**10.5**	11.8	NS
**Immulectin-2**	66,0	**-1.9**	ND	22.1	**26.9**	31.8	2.7	**6.0**	9.3	20.9	**25.0**	29.0	**-4.5**	NS	20.0	**25.9**	31.7	3.2	**7.6**	12.0	**-3.4**
**Lysozyme**	4,0	**-2.2**	ND	36.0	**46.8**	57.6	8.2	**18.7**	29.1	55.4	**79.9**	104.3	**-2.3**	**+1.7**	22.7	**31.8**	40.8	32.4	**44.7**	57.0	NS
**Calreticulin**	NS	**-2.0**	ND	5,5	**7,4**	9,2	1,1	**2,4**	3,8	3.5	**5.5**	7.5	**-3,0**	NS	3.5	**4.3**	5.1	3.7	**4.6**	5.4	NS

**FAT BODY**																					

**Lysozyme**	--	NS	**+1.7**	9.9	**11.8**	13.8	10.9	**11.7**	12.5	39.6	**86.7**	133.8	NS	**+7.3**	33.8	**35.7**	37.6	45.5	**56.3**	67.1	**+1.6**
**Calreticulin**	--	**-2.6**	NS	6.5	**7.7**	8.9	2.0	**2.5**	2.9	6.5	**7.6**	8.6	**-2.9**	NS	4.5	**5.8**	7.2	4.8	**5.8**	6.8	NS

Variations in gene transcript levels measured by quantitative RT-PCR in response to virus infection. Calculated mean of the relative cDNA starting quantity (N0) and consequential fold changes (V/C: virus/control) for selected *Spodoptera frugiperda *genes in control and virus-infected hemocytes and fat body 24 hours after injection. Minimum and maximum values obtained from the 18 or 24 replicates are given in addition to the mean N0 value. For HdIV assays, the control consisted in injection of PBS, for JcDNV (DNV), in injection of healthy *S. frugiperda *larvae crushed in PBS; HdIV95 corresponds to injections of heat-inactivated HdIV. H/F: ratio of the relative cDNA starting quantity (N0) between hemocytes and fat body in control PBS samples. ND: not done; NS: not significant; fold change threshold at 1.5 for both microarray and quantitative RT-PCR analyses, with FDR median and 90th percentiles at 0%.

**Table 3:** Variation in SR-C gene transcript levels measured by quantitative RT-PCR.

**HEMOCYTES (100 ng)**							**HEMOCYTES (4 ng)**						
**mean N0**						**Fold**	**mean N0**						**Fold**
**control (n = 9)**			**beads (n = 9)**			**B/C**	**control (n = 9)**			**beads (n = 9)**			**B/C**
min	mean	max	min	mean	max		min	mean	max	min	mean	max	
36.1	**54.6**	73.1	195.3	**220.5**	245.7	**+4.0**	7.8	**11.6**	15.3	20.9	**24.6**	28.2	**+2.1**

Variation in SR-C gene transcript levels measured by quantitative RT-PCR in response to injection of Sephadex beads in the hemocyte population collected 6 to 7 hours after injection. Calculated mean of the relative cDNA starting quantity (N0) and consequential fold changes (B/C: beads/control). Fold change threshold at 1.5 for quantitative RT-PCR analyses, with FDR median and 90th percentiles at 0%.

## References

[B1] Hoffmann JA (2003). The immune response of Drosophila. Nature.

[B2] Lavine MD, Strand MR (2002). Insect hemocytes and their role in immunity. Insect Biochem Mol Biol.

[B3] Hoffmann JA (1995). Innate immunity of insects. Curr Opin Immunol.

[B4] Hultmark D (2003). Drosophila immunity: paths and patterns. Curr Opin Immunol.

[B5] Kanost MR, Jiang H, Yu XQ (2004). Innate immune responses of a lepidopteran insect, Manduca sexta. Immunol Rev.

[B6] Leclerc V, Reichhart JM (2004). The immune response of Drosophila melanogaster. Immunol Rev.

[B7] Gillespie JP, Kanost MR, Trenczek T (1997). Biological mediators of insect immunity. Annu Rev Entomol.

[B8] Dostert C, Jouanguy E, Irving P, Troxler L, Galiana-Arnoux D, Hetru C, Hoffmann JA, Imler JL (2005). The Jak-STAT signaling pathway is required but not sufficient for the antiviral response of drosophila. Nat Immunol.

[B9] Roxstrom-Lindquist K, Terenius O, Faye I (2004). Parasite-specific immune response in adult Drosophila melanogaster: a genomic study. EMBO Rep.

[B10] Zambon RA, Nandakumar M, Vakharia VN, Wu LP (2005). The Toll pathway is important for an antiviral response in Drosophila. Proc Natl Acad Sci U S A.

[B11] Furukawa S, Tanaka H, Nakazawa H, Ishibashi J, Shono T, Yamakawa M (1999). Inducible gene expression of moricin, a unique antibacterial peptide from the silkworm (Bombyx mori). Biochem J.

[B12] Volkoff AN, Rocher J, d'Alencon E, Bouton M, Landais I, Quesada-Moraga E, Vey A, Fournier P, Mita K, Devauchelle G (2003). Characterization and transcriptional profiles of three Spodoptera frugiperda genes encoding cysteine-rich peptides. A new class of defensin-like genes from lepidopteran insects?. Gene.

[B13] Yamakawa M, Tanaka H (1999). Immune proteins and their gene expression in the silkworm, Bombyx mori. Dev Comp Immunol.

[B14] Lavine MD, Chen G, Strand MR (2005). Immune challenge differentially affects transcript abundance of three antimicrobial peptides in hemocytes from the moth Pseudoplusia includens. Insect Biochem Mol Biol.

[B15] Yu XQ, Zhu YF, Ma C, Fabrick JA, Kanost MR (2002). Pattern recognition proteins in Manduca sexta plasma. Insect Biochem Mol Biol.

[B16] Popham HJ, Shelby KS, Brandt SL, Coudron TA (2004). Potent virucidal activity in larval Heliothis virescens plasma against Helicoverpa zea single capsid nucleopolyhedrovirus. J Gen Virol.

[B17] Hirai M, Terenius O, Li W, Faye I (2004). Baculovirus and dsRNA induce Hemolin, but no antibacterial activity, in Antheraea pernyi. Insect Mol Biol.

[B18] Washburn JO, Haas-Stapleton EJ, Tan FF, Beckage NE, Volkman LE (2000). Co-infection of Manduca sexta larvae with polydnavirus from Cotesia congregata increases susceptibility to fatal infection by Autographa californica M Nucleopolyhedrovirus. J Insect Physiol.

[B19] Lavine MD, Strand MR (2003). Haemocytes from Pseudoplusia includens express multiple alpha and beta integrin subunits. Insect Mol Biol.

[B20] Nardi JB, Zhuang S, Pilas B, Bee CM, Kanost MR (2005). Clustering of adhesion receptors following exposure of insect blood cells to foreign surfaces. Journal Insect Physiology.

[B21] Pech LL, Strand MR (2000). Plasmatocytes from the moth Pseudoplusia includens induce apoptosis of granular cells. J Insect Physiol.

[B22] Schmidt O, Theopold U, Strand M (2001). Innate immunity and its evasion and suppression by hymenopteran endoparasitoids. Bioessays.

[B23] Ribeiro C, Brehelin M (2006). Insect haemocytes: What type of cell is that?. J Insect Physiol.

[B24] Glatz RV, Asgari S, Schmidt O (2004). Evolution of polydnaviruses as insect immune suppressors. Trends Microbiol.

[B25] Labrosse C, Carton Y, Dubuffet A, Drezen JM, Poirie M (2003). Active suppression of D. melanogaster immune response by long gland products of the parasitic wasp Leptopilina boulardi. J Insect Physiol.

[B26] Labrosse C, Eslin P, Doury G, Drezen JM, Poirie M (2005). Haemocyte changes in D. melanogaster in response to long gland components of the parasitoid wasp Leptopilina boulardi: a Rho-GAP protein as an important factor. J Insect Physiol.

[B27] Labrosse C, Stasiak K, Lesobre J, Grangeia A, Huguet E, Drezen JM, Poirie M (2005). A RhoGAP protein as a main immune suppressive factor in the Leptopilina boulardi (Hymenoptera, Figitidae)-Drosophila melanogaster interaction. Insect Biochem Mol Biol.

[B28] Pennacchio F, Strand MR (2006). Evolution of developmental strategies in parasitic hymenoptera. Annu Rev Entomol.

[B29] Volkoff AN, Ravallec M, Bossy JP, Cerutti P, Rocher J, Cerutti M, Devauchelle G (1995). The replication of Hyposoter didymator polydnavirus: Cytopathology of the calyx cells in the parasitoid. Biology of the Cell.

[B30] Galibert L, Rocher J, Ravallec M, Duonor-Cerutti M, Webb BA, Volkoff AN (2003). Two Hyposoter didymator ichnovirus genes expressed in the lepidopteran host encode secreted or membrane-associated serine and threonine rich proteins in segments that may be nested. J Insect Physiol.

[B31] Volkoff AN, Beliveau C, Rocher J, Hilgarth R, Levasseur A, Duonor-Cerutti M, Cusson M, Webb BA (2002). Evidence for a conserved polydnavirus gene family: ichnovirus homologs of the CsIV repeat element genes. Virology.

[B32] Volkoff AN, Cerutti P, Rocher J, Ohresser MC, Devauchelle G, Duonor-Cerutti M (1999). Related RNAs in lepidopteran cells after in vitro infection with Hyposoter didymator virus define a new polydnavirus gene family. Virology.

[B33] Volkoff AN, Rocher J, Cerutti P, Ohresser MC, d'Aubenton-Carafa Y, Devauchelle G, Duonor-Cerutti M (2001). Persistent expression of a newly characterized Hyposoter didymator polydnavirus gene in long-term infected lepidopteran cell lines. J Gen Virol.

[B34] Amaya KE, Asgari S, Jung R, Hongskula M, Beckage NE (2005). Parasitization of Manduca sexta larvae by the parasitoid wasp Cotesia congregata induces an impaired host immune response. J Insect Physiol.

[B35] Turnbull MW, Martin SB, Webb BA (2004). Quantitative analysis of hemocyte morphological abnormalities associated with *Campoletis sonorensis *parasitization. J Insect Sci.

[B36] Asgari S (2006). Venom proteins from polydnavirus-producing endoparasitoids: their role in host-parasite interactions. Arch Insect Biochem Physiol.

[B37] Bae S, Kim Y (2004). Host physiological changes due to parasitism of a braconid wasp, Cotesia plutellae, on diamondback moth, Plutella xylostella. Comp Biochem Physiol A Mol Integr Physiol.

[B38] Beckage NE (1998). Modulation of immune responses to parasitoids by polydnaviruses. Parasitology.

[B39] Shelby KS, Adeyeye OA, Okot-Kotber BM, Webb BA (2000). Parasitism-linked block of host plasma melanization. J Invertebr Pathol.

[B40] Shelby KS, Webb BA (1999). Polydnavirus-mediated suppression of insect immunity. J Insect Physiol.

[B41] Stoltz DB, Cook DI (1983). Inhibition of host phenoloxydase activity by parasitoid hymenoptera. Experientia.

[B42] Cerenius L, Soderhall K (2004). The prophenoloxidase-activating system in invertebrates. Immunol Rev.

[B43] Espagne E, Dupuy C, Huguet E, Cattolico L, Provost B, Martins N, Poirie M, Periquet G, Drezen JM (2004). Genome sequence of a polydnavirus: insights into symbiotic virus evolution. Science.

[B44] Kroemer JA, Webb BA (2004). Polydnavirus genes and genomes: emerging gene families and new insights into polydnavirus replication. Annu Rev Entomol.

[B45] Webb BA, Strand MR, Dickey SE, Beck MH, Hilgarth RS, Barney WE, Kadash K, Kroemer JA, Lindstrom KG, Rattanadechakul W, Shelby KS, Thoetkiattikul H, Turnbull MW, Witherell RA (2006). Polydnavirus genomes reflect their dual roles as mutualists and pathogens. Virology.

[B46] Kroemer JA, Webb BA (2005). Ikappabeta-related vankyrin genes in the Campoletis sonorensis ichnovirus: temporal and tissue-specific patterns of expression in parasitized Heliothis virescens lepidopteran hosts. J Virol.

[B47] Thoetkiattikul H, Beck MH, Strand MR (2005). Inhibitor kappaB-like proteins from a polydnavirus inhibit NF-kappaB activation and suppress the insect immune response. Proc Natl Acad Sci U S A.

[B48] Turnbull MW, Volkoff AN, Webb BA, Phelan P (2005). Functional gap junction genes are encoded by insect viruses. Curr Biol.

[B49] Asgari S, Schmidt O (2004). Isolation of an imaginal disc growth factor homologue from Pieris rapae and its expression following parasitization by Cotesia rubecula. J Insect Physiol.

[B50] Consoli FL, Brandt SL, Coudron TA, Vinson SB (2005). Host regulation and release of parasitism-specific proteins in the system Toxoneuron nigriceps-Heliothis virescens. Comp Biochem Physiol B Biochem Mol Biol.

[B51] Dong K, Zhang D, Dahlman DL (1996). Down-regulation of juvenile hormone esterase and arylphorin production in *Heliothis virescens *larvae parasitized by *Microplitis croceipes*. Archives of Insect Biochemistry and Physiology.

[B52] Kim Y (2005). Identification of host translation inhibitory factor of Campoletis sonorensis ichnovirus on the tobacco budworm, Heliothis virescens. Arch Insect Biochem Physiol.

[B53] Shelby KS, Cui L, Webb BA (1998). Polydnavirus-mediated inhibition of lysozyme gene expression and the antibacterial response. Insect Mol Biol.

[B54] Shelby KS, Webb BA (1994). Polydnavirus infection inhibits synthesis of an insect plasma protein, arylphorin. J Gen Virol.

[B55] Shelby KS, Webb BA (1997). Polydnavirus infection inhibits translation of specific growth-associated host proteins. Insect Biochem Mol Biol.

[B56] De Gregorio E, Spellman PT, Rubin GM, Lemaitre B (2001). Genome-wide analysis of the Drosophila immune response by using oligonucleotide microarrays. Proc Natl Acad Sci U S A.

[B57] Johansson KC, Metzendorf C, Soderhall K (2005). Microarray analysis of immune challenged Drosophila hemocytes. Exp Cell Res.

[B58] Aguilar R, Jedlicka AE, Mintz M, Mahairaki V, Scott AL, Dimopoulos G (2005). Global gene expression analysis of Anopheles gambiae responses to microbial challenge. Insect Biochem Mol Biol.

[B59] Wertheim B, Kraaijeveld AR, Schuster E, Blanc E, Hopkins M, Pletcher SD, Strand MR, Partridge L, Godfray HC (2005). Genome-wide gene expression in response to parasitoid attack in Drosophila. Genome Biol.

[B60] Dimopoulos G, Christophides GK, Meister S, Schultz J, White KP, Barillas-Mury C, Kafatos FC (2002). Genome expression analysis of Anopheles gambiae: responses to injury, bacterial challenge, and malaria infection. Proc Natl Acad Sci U S A.

[B61] http://bioweb.ensam.inra.fr/spodobase/.

[B62] Bergoin M, Tijssen P (1998). Biological and molecular properties of Densoviruses and their use in protein expression and biological control.

[B63] Bachali S, Jager M, Hassanin A, Schoentgen F, Jolles P, Fiala-Medioni A, Deutsch JS (2002). Phylogenetic analysis of invertebrate lysozymes and the evolution of lysozyme function. J Mol Evol.

[B64] Gorman MJ, Kankanala P, Kanost MR (2004). Bacterial challenge stimulates innate immune responses in extra-embryonic tissues of tobacco hornworm eggs. Insect Mol Biol.

[B65] Sun SC, Asling B, Faye I (1991). Organization and expression of the immunoresponsive lysozyme gene in the giant silk moth, Hyalophora cecropia. J Biol Chem.

[B66] Jiang H, Wang Y, Kanost MR (1998). Pro-phenol oxidase activating proteinase from an insect, Manduca sexta: a bacteria-inducible protein similar to Drosophila easter. Proc Natl Acad Sci U S A.

[B67] Jiang H, Kanost MR (2000). The clip-domain family of serine proteinases in arthropods. Insect Biochem Mol Biol.

[B68] Zou Z, Wang Y, Jiang H (2005). Manduca sexta prophenoloxidase activating proteinase-1 (PAP-1) gene: organization, expression, and regulation by immune and hormonal signals. Insect Biochem Mol Biol.

[B69] Yu XQ, Kanost MR (2000). Immulectin-2, a lipopolysaccharide-specific lectin from an insect, Manduca sexta, is induced in response to gram-negative bacteria. J Biol Chem.

[B70] Koizumi N, Imamura M, Kadotani T, Yaoi K, Iwahana H, Sato R (1999). The lipopolysaccharide-binding protein participating in hemocyte nodule formation in the silkworm Bombyx mori is a novel member of the C-type lectin superfamily with two different tandem carbohydrate-recognition domains. FEBS Lett.

[B71] Ling E, Yu XQ (2006). Cellular encapsulation and melanization are enhanced by immulectins, pattern recognition receptors from the tobacco hornworm Manduca sexta. Dev Comp Immunol.

[B72] Yu XQ, Tracy ME, Ling E, Scholz FR, Trenczek T (2005). A novel C-type immulectin-3 from Manduca sexta is translocated from hemolymph into the cytoplasm of hemocytes. Insect Biochem Mol Biol.

[B73] Zhu Y, Johnson TJ, Myers AA, Kanost MR (2003). Identification by subtractive suppression hybridization of bacteria-induced genes expressed in Manduca sexta fat body. Insect Biochem Mol Biol.

[B74] Nakhasi HL, Pogue GP, Duncan RC, Joshi M, Atreya CD, Lee NS, Dwyer DM (1998). Implications of calreticulin function in parasite biology. Parasitology Today.

[B75] Gelebart P, Opas M, Michalak M (2005). Calreticulin, a Ca2+-binding chaperone of the endoplasmic reticulum. Int J Biochem Cell Biol.

[B76] Johnson S, Michalak M, Opas M, Eggleton P (2001). The ins and outs of calreticulin: from the ER lumen to the extracellular space. Trends Cell Biol.

[B77] Gardai SJ, McPhillips KA, Frasch SC, Janssen WJ, Starefeldt A, Murphy-Ullrich JE, Bratton DL, Oldenborg PA, Michalak M, Henson PM (2005). Cell-surface calreticulin initiates clearance of viable or apoptotic cells through trans-activation of LRP on the phagocyte. Cell.

[B78] Choi JY, Whitten MM, Cho MY, Lee KY, Kim MS, Ratcliffe NA, Lee BL (2002). Calreticulin enriched as an early-stage encapsulation protein in wax moth Galleria mellonella larvae. Dev Comp Immunol.

[B79] Asgari S, Schmidt O (2003). Is cell surface calreticulin involved in phagocytosis by insect hemocytes?. J Insect Physiol.

[B80] Zhang G, Schmidt O, Asgari S (2005). A calreticulin-like protein from endoparasitoid venom fluid is involved in host hemocyte inactivation. Dev Comp Immunol.

[B81] Ramet M, Pearson A, Manfruelli P, Li X, Koziel H, Gobel V, Chung E, Krieger M, Ezekowitz RA (2001). Drosophila scavenger receptor CI is a pattern recognition receptor for bacteria. Immunity.

[B82] Costa SC, Ribeiro C, Girard PA, Zumbihl R, Brehelin M (2005). Modes of phagocytosis of Gram-positive and Gram-negative bacteria by Spodoptera littoralis granular haemocytes. J Insect Physiol.

[B83] Lazzaro BP (2005). Elevated polymorphism and divergence in the class C scavenger receptors of Drosophila melanogaster and D. simulans. Genetics.

[B84] Niki I, Yokokura H, Sudo T, Kato M, Hidaka H (1996). Ca2+ signaling and intracellular Ca2+ binding proteins. J Biochem (Tokyo).

[B85] Kotani E, Yamakawa M, Iwamoto S, Tashiro M, Mori H, Sumida M, Matsubara F, Taniai K, Kadono-Okuda K, Kato Y, Mori H (1995). Cloning and expression of the gene of hemocytin, an insect humoral lectin which is homologous with the mammalian von Willebrand factor. Biochim Biophys Acta.

[B86] Cooper DN (2002). Galectinomics: finding themes in complexity. Biochim Biophys Acta.

[B87] Hughes RC (2001). Galectins as modulators of cell adhesion. Biochimie.

[B88] Liu FT, Patterson RJ, Wang JL (2002). Intracellular functions of galectins. Biochim Biophys Acta.

[B89] Pace KE, Baum LG (2004). Insect galectins: roles in immunity and development. Glycoconj J.

[B90] Christophides GK, Vlachou D, Kafatos FC (2004). Comparative and functional genomics of the innate immune system in the malaria vector Anopheles gambiae. Immunol Rev.

[B91] Lavine MD, Beckage NE (1995). Polydnaviruses: potent mediators of host insect immune dysfunction. Parasitol Today.

[B92] Le NT, Asgari S, Amaya K, Tan FF, Beckage NE (2003). Persistence and expression of Cotesia congregata polydnavirus in host larvae of the tobacco hornworm, Manduca sexta. J Insect Physiol.

[B93] Leclerc V, Pelte N, El Chamy L, Martinelli C, Ligoxygakis P, Hoffmann JA, Reichhart JM (2006). Prophenoloxidase activation is not required for survival to microbial infections in Drosophila. EMBO Rep.

[B94] Huang LH, Christensen BM, Chen CC (2001). Molecular cloning of a second prophenoloxidase cDNA from the mosquito Armigeres subalbatus: prophenoloxidase expression in blood-fed and microfilariae-inoculated mosquitoes. Insect Mol Biol.

[B95] Rajagopal R, Thamilarasi K, Venkatesh GR, Srinivas P, Bhatnagar RK (2005). Immune cascade of Spodoptera litura: cloning, expression, and characterization of inducible prophenol oxidase. Biochem Biophys Res Commun.

[B96] Strand MR, Pech LL (1995). Microplitis demolitor polydnavirus induces apoptosis of a specific haemocyte morphotype in Pseudoplusia includens. J Gen Virol.

[B97] Beckage NE, Tan FF, Schleifer KW, Lane RD, Cherubin LL (1994). Characterization and biological effects of *Cotesia congregata *polydnavirus on host larvae of the tobacco hornworm, *Manduca sexta*. Arch Insect Biochem Physiol.

[B98] Matsumoto Y, Oda Y, Uryu M, Hayakawa Y (2003). Insect cytokine growth-blocking peptide triggers a termination system of cellular immunity by inducing its binding protein. J Biol Chem.

[B99] Strand MR, Hayakawa Y, Clark KD (2000). Plasmatocyte spreading peptide (PSP1) and growth blocking peptide (GBP) are multifunctional homologs. J Insect Physiol.

[B100] Irving P, Ubeda JM, Doucet D, Troxler L, Lagueux M, Zachary D, Hoffmann JA, Hetru C, Meister M (2005). New insights into Drosophila larval haemocyte functions through genome-wide analysis. Cell Microbiol.

[B101] Negre V, Hotelier T, Volkoff A-N, Gimenez S, Cousserans F, Mita K, Sabau X, Rocher J, López-Ferber M, d'Alençon E, Audant P, Sabourault C, Bidegainberry V, Hilliou F, Fournier P SPODOBASE : an EST database for the lepidopteran crop pest Spodoptera. BMC Bioinformatics.

[B102] Hugot K, Riviere MP, Moreilhon C, Dayem MA, Cozzitorto J, Arbiol G, Barbry P, Weiss C, Galiana E (2004). Coordinated regulation of genes for secretion in tobacco at late developmental stages: association with resistance against oomycetes. Plant Physiol.

[B103] Rivers CF, Longworth JF (1972). A non-occluded virus of Junonia coenia (Nymphalidae : Lepidotera). Journal of Invertebrate Pathology.

[B104] http://www.prontosystems.com/.

[B105] http://cardioserve.nantes.inserm.fr/mad/madscan/.

[B106] Yang YH, Dudoit S, Luu P, Lin DM, Peng V, Ngai J, Speed TP (2002). Normalization for cDNA microarray data: a robust composite method addressing single and multiple slide systematic variation. Nucleic Acids Res.

[B107] Le Meur N, Lamirault G, Bihouee A, Steenman M, Bedrine-Ferran H, Teusan R, Ramstein G, Leger JJ (2004). A dynamic, web-accessible resource to process raw microarray scan data into consolidated gene expression values: importance of replication. Nucleic Acids Res.

[B108] http://www-stat.stanford.edu/~tibs/SAM/.

[B109] Didier G, Brezellec P, Remy E, Henaut A (2002). GeneANOVA – gene expression analysis of variance. Bioinformatics.

[B110] Tusher VG, Tibshirani R, Chu G (2001). Significance analysis of microarrays applied to the ionizing radiation response. Proc Natl Acad Sci U S A.

[B111] Yang IV, Chen E, Hasseman JP, Liang W, Frank BC, Wang S, Sharov V, Saeed AI, White J, Li J, Lee NH, Yeatman TJ, Quackenbush J (2002). Within the fold: assessing differential expression measures and reproducibility in microarray assays. Genome Biol.

[B112] Ramakers C, Ruijter JM, Deprez RH, Moorman AF (2003). Assumption-free analysis of quantitative real-time polymerase chain reaction (PCR) data. Neurosci Lett.

[B113] Vandesompele J, De Preter K, Pattyn F, Poppe B, Van Roy N, De Paepe A, Speleman F (2002). Accurate normalization of real-time quantitative RT-PCR data by geometric averaging of multiple internal control genes. Genome Biol.

[B114] Shida K, Terajima D, Uchino R, Ikawa S, Ikeda M, Asano K, Watanabe T, Azumi K, Nonaka M, Satou Y, Satoh N, Satake M, Kawazoe Y, Kasuya A (2003). Hemocytes of Ciona intestinalis express multiple genes involved in innate immune host defense. Biochem Biophys Res Commun.

[B115] Schmit AR, Ratcliffe NA (1977). The encapsulation of foreign tissue implants in Galleria mellonella larvae. J Insect Physiol.

