# Supplementary material for: Gene expression profiling of Spodoptera frugiperda hemocytes and fat body using cDNA microarray reveals polydnavirus-associated variations in lepidopteran host genes transcript levels
Source: BMC Genomics. 2006 Jun 21;7:160. doi: 10.1186/1471-2164-7-160 (PMC1559612; doi:10.1186/1471-2164-7-160)
Supplement: Additional file 1 — Complementary results of microarray analysis.1A: Results obtained for the HdIV cDNAs spotted on the microarray. Results obtained for the HdIV cDNAs spotted on the microarray, in the hemocytes (H) and in the fat body (FB) 24 hours after injection of HdIV, as detected by a modified t-test from the SAM package and an ANOVA based microarray analysis, GeneANOVA. Only the genes with a fold change superior or equal to 1.5 with both a "false discovery rate" (FDR) median and 90th percentiles of 0% were kept. The SAM fold and q-value (the lowest FDR at which the gene is called significant) as well as the ANOVA F "gene-condition" value and the p-value are given. Gene Name: annotation of the sequence. (B) List of additional clones corresponding to each gene. List of additional cDNA clones that have been spotted on the microarray and which corresponded to the same gene. [file 1471-2164-7-160-S1.doc]

**Additional file 1:** Complementary results of microarray analysis.

**1A:** Results obtained for the HdIV cDNAs spotted on the microarray.

**1B:** List of additional clones corresponding to each gene.

- PO1, 4 clones Sf1H00908-5-1, Sf1H00902-5-1, Sf1H00555-5-1, Sf1H00689-5-1
- PO2, 3 clones, Sf1H00508-5-1, Sf1H00350-5-1, Sf1H02647-5-1,
- Scavenger receptor iso-1, 2 clones, Sf1H00106-5-1 Sf1H02666-5-1
- Hemicentin, 3 clones, Sf1H01096-5-1, Sf1H02093-5-1, Sf1H01698-5-1
- Lysozyme, 2 clones, Sf1H00171-5-1, Sf1H00171-5-1
- collagen alpha 1 (IV) , 2 clones, Sf9L07414, Sf1H00310-5-1
- Hypothetical protein *A. gambiae*, 5 clones, Sf1H02709-3-1, Sf1H00730-5-1, Sf1H00695-5-1, Sf1H03044-5-1, Sf1H02372-3-1
- similar to LPS-induced TNF-alpha factor, 2 clones Sf1H00362-5-1, Sf1H00681-5-1
- similar to pancreatic triacylglyceride lipase, 2 clones, Sf1H00304-5-1, Sf1H03038-5-1
- lysozyme, 2 clones, Sf1H01136-5-1, Sf1H00171-5-1
