# Supplementary material for: Gene expression profiling of Spodoptera frugiperda hemocytes and fat body using cDNA microarray reveals polydnavirus-associated variations in lepidopteran host genes transcript levels
Source: BMC Genomics. 2006 Jun 21;7:160. doi: 10.1186/1471-2164-7-160 (PMC1559612; doi:10.1186/1471-2164-7-160)
Supplement: Additional file 2 — Functional Spodoptera frugiperda gene classification. Functional gene classification based on the one described in Shida et al. for the ascidian Ciona intestinalis [114], and used for the annotation of the Spodoptera frugiperda sequences (Volkoff et al., in prep.). [file 1471-2164-7-160-S2.doc]

**Additional file 2:** Functional *Spodoptera frugiperda* gene classification.

**A: FUNCTIONS THAT MANY KINDS OF CELLS USE**

AI transportation and binding proteins for ions and small molecules

AII RNA processing proteins and enzymes

AIII cell replication, histones, DNA modifications

AIV cytoskeleton, cell shape and membrane proteins

AV protein synthesis, ribosomal proteins

AVI intermediary synthesis, catabolism enzymes

AVII stress response, detoxification and cell defense proteins

AVIII protein degradation and processing

AIX transport and binding proteins for proteins and macromolecules

**B: CELL-CELL COMMUNICATION**

BI signaling receptors and ligands

BII intracellular signal transduction pathway molecules

BIII extracellular matrix proteins and cell adhesion

**C: TRANSCRIPTION FACTORS and other gene regulatory proteins**

**D: MOLECULES DESCRIBED IN INSECTS**

DI defense molecules

DII storage molecules

DIII metabolism and other proteins of unknown function

DIV proteases (secreted)

**E: OTHERS**

EI similar to known protein but not enough information to classify

EII similar to hypothetical proteins

EIII no significant similarity with known proteins (treshold e-10)
