# Supplementary material for: Gene expression profiling of Spodoptera frugiperda hemocytes and fat body using cDNA microarray reveals polydnavirus-associated variations in lepidopteran host genes transcript levels
Source: BMC Genomics. 2006 Jun 21;7:160. doi: 10.1186/1471-2164-7-160 (PMC1559612; doi:10.1186/1471-2164-7-160)
Supplement: Additional file 3 — List of the primers designed for quantitative RT-PCR analysis. List of the forward and reverse primers designed for quantitative RT-PCR analysis of the 8 selected genes and the 3 endogenous reference genes. [file 1471-2164-7-160-S3.doc]

**Additional file 3:** List of the primers designed for quantitative RT-PCR analysis.
